# *Porphyromonas gingivalis* FimA Fimbriae: Fimbrial Assembly by *fimA* Alone in the *fim* Gene Cluster and Differential Antigenicity among *fimA* Genotypes

**DOI:** 10.1371/journal.pone.0043722

**Published:** 2012-09-07

**Authors:** Keiji Nagano, Yoshiaki Hasegawa, Yuki Abiko, Yasuo Yoshida, Yukitaka Murakami, Fuminobu Yoshimura

**Affiliations:** 1 Department of Microbiology, School of Dentistry, Aichi Gakuin University, Nagoya, Japan; 2 Division of Oral Infections and Health Sciences, Department of Oral Microbiology, School of Dentistry, Asahi University, Gifu, Japan; University of Illinois at Urbana-Champaign, United States of America

## Abstract

The periodontal pathogen *Porphyromonas gingivalis* colonizes largely through FimA fimbriae, composed of polymerized FimA encoded by *fimA*. *fimA* exists as a single copy within the *fim* gene cluster (*fim* cluster), which consists of seven genes: *fimX*, *pgmA* and *fimA-E*. Using an expression vector, *fimA* alone was inserted into a mutant from which the whole *fim* cluster was deleted, and the resultant complement exhibited a fimbrial structure. Thus, the genes of the *fim* cluster other than *fimA* were not essential for the assembly of FimA fimbriae, although they were reported to influence FimA protein expression. It is known that there are various genotypes for *fimA*, and it was indicated that the genotype was related to the morphological features of FimA fimbriae, especially the length, and to the pathogenicity of the bacterium. We next complemented the *fim* cluster-deletion mutant with *fimA* genes cloned from *P. gingivalis* strains including genotypes I to V. All genotypes showed a long fimbrial structure, indicating that FimA itself had nothing to do with regulation of the fimbrial length. In FimA fimbriae purified from the complemented strains, types I, II, and III showed slightly higher thermostability than types IV and V. Antisera of mice immunized with each purified fimbria principally recognized the polymeric, structural conformation of the fimbriae, and showed low cross-reactivity among genotypes, indicating that FimA fimbriae of each genotype were antigenically different. Additionally, the activity of a macrophage cell line stimulated with the purified fimbriae was much lower than that induced by *Escherichia coli* lipopolysaccharide.

## Introduction

*Porphyromonas gingivalis*, a gram-negative anaerobe, is thought to be a major causative pathogen of periodontal diseases [1]. The pathogen colonizes subgingival sites as a biofilm associate. Biofilm formation of *P. gingivalis* is mediated largely through fimbriae, filamentous structures on the cell surface. *P. gingivalis* generally expresses two distinct fimbriae, called FimA and Mfa1 fimbriae, which are composed of polymerized FimA and Mfa1 proteins encoded by the *fimA* and *mfa1* genes, respectively [2]. Several accessory components are also associated as minor subunits with both fimbriae.

**Figure 1:**
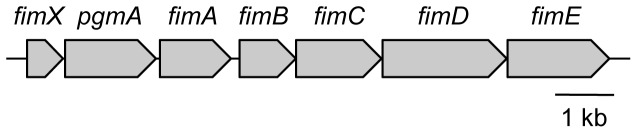
*P. gingivalis fim* gene cluster. We constructed a mutant with the whole region of the *fim* gene cluster from *fimX* through *fimE* deleted. The schema was drawn on the basis of ATCC 33277. However, genome-analyzed strains of W83 and TDC60 show that they are the same as that of ATCC 33277 in the gene arrangement.

FimA fimbriae in *P. gingivalis* were discovered over 30 years ago and have been intensively studied [2]. Dickinson *et al.* [3], Watanabe *et al*. [4], and genome analyses [5], [6], [7] have revealed that *fimA* exists as a single copy within the *fim* gene cluster (*fim* cluster), consisting of seven genes, *fimX*, *pgmA* and *fimA-E* (Fig. 1), encoding FimX, PgmA and FimA-E proteins, respectively. It is known that *P. gingivalis* strains 381 and ATCC 33277 (33277) express aberrant long FimA fimbriae over a few micrometers in length, and we demonstrated that this was attributable to FimB deficiency [8]. Restoration of FimB in 33277 shortened the fimbriae, indicating that FimB regulates FimA fimbrial length. FimC, FimD and FimE associate with the FimA fimbriae as accessory components [9], [10], and it has been suggested that they play an important role in adhesion [10]. Even when FimB-E were deficient, FimA protein was produced and polymerized to form the fimbrial structure, although the amount of fimbriae decreased [10], [11]. The upstream gene *fimX* was reported to lead drastic reduction in *fimA* transcription [12], whereas a mutation in *pgmA* considerably decreased it [12], indicating a principal role for them in the regulation cascade of FimA protein expression. PgmA exists in the outer membrane [13], but FimX has not been fully investigated. In this study, we examined the *fim* cluster, especially *fimX* and *pgmA*, focusing on their functions in fimbrial polymerization and elongation. However, we found that *fimX* and *pgmA* were not essential for FimA polymerization, and *fimA* of the *fim* cluster solely conferred the ability for fimbrial formation.

It is known that there are six genotypes of *fimA*, types I–V and Ib [14], and that the genotype is related to the morphological features of FimA fimbriae [15], [16] and virulence of *P. gingivalis* strains [17], [18]. However, this is still controversial because some argue that the virulence is not related to specific genotypes of the organism [19], [20]. Others indicated the drawbacks of the genotyping methods used [21], [22], [23]. To understand more exactly their basic features, we purified FimA fimbriae from strains with each genotype, and analyzed them by biochemical and immunological methods.

## Materials and Methods

### P. gingivalis strains and culture conditions

The *P. gingivalis* the wild-type strains used here included five genotypes of *fimA* [14]: type I, 33277; type II, TDC60; type III, 6/26; type IV, W83 and HG564; and type V, HNA99. Whole-genome sequences have been published for 33277 [6], TDC60 [7] and W83 [5], whereas partial sequences around *fimA* were published for 6/26 (GenBank GI: 456504), HG564 (GI: 456506) and HNA99 (GI: 6429668). *P. gingivalis* was cultivated in Modified GAM medium (Nissui Pharmaceutical Co., Ltd, Tokyo, Japan), supplemented with 5% laked rabbit blood for agar plate, at 37°C under anaerobic conditions. When necessary, the following antibiotics were added: 10 µg/ml chloramphenicol, 10 µg/ml erythromycin and 1 µg/ml tetracycline.

### DNA sequencing

A purified PCR product and plasmid DNA were used as templates for the DNA cycle sequencing with a BigDye Terminator v3.1 Cycle Sequencing kit (Applied Biosystems, Foster City, CA). The products of the DNA cycle sequencing reaction were purified and analyzed using a 3130 Genetic Analyzer (Applied Biosystems).

### Construction of mfa1- and fim cluster-deletion mutant

Primers used (Tables S1 and S2) and schemes (Figs. S1 and S2) for mutant construction are shown in the supplemental material. Here we briefly describe the construction methods. We constructed a *fim* cluster-deletion mutant from two *P. gingivalis* strains, W83 and an *mfa1*-deletion mutant of 33277 (33277 Δ*mfa1*). 33277 expresses both FimA and Mfa1 fimbriae well. To avoid confusion with Mfa1 fimbriae, we first constructed 33277 Δ*mfa1* from 33277 by replacing *mfa1* with the chloramphenicol acetyltransferase gene (*cat*) (Fig. S1) as previously described [24]. Since W83 did not express Mfa1 fimbriae, we used W83 without deletion of *mfa1*. Although W83 rarely expresses FimA fimbriae because of transcriptional inactivity [25], it possesses a *fim* cluster similar to that of 33277. Therefore, we deleted the *fim* cluster from W83 to interpret the results simply. The whole region from *fimX* to *fimE* (Fig. 1) was replaced with the erythromycin resistance cassette isolated from pVA2198 [26] in 33277 Δ*mfa1* and W83 (Fig. S2). We hereafter call the resulting mutants as 33277 Δ*mfa1* Δ*fim* cluster and W83 Δ*fim* cluster.

### Introduction of genes into *P. gingivalis* using an expression vector

We used pT-COW with the *ragA* promoter (pT-COW::*ragAP*) incorporated to express target genes in *P. gingivalis* [27], [28]. Primers used (Tables S3 and S4) and schemes (Figs. S3 and S4) for construction are shown in the supplemental material. Briefly, target genes were amplified by PCR using primers with restriction enzyme recognition sites incorporated. PCR products were digested by the restriction enzymes, and they were inserted into pT-COW::*ragAP* digested by the same enzymes. After confirming that there was no unintended mutation in the target genes by DNA sequencing, they were introduced into the 33277 Δ*mfa1* Δ*fim* cluster and W83 Δ*fim* cluster.

### Preparation of whole-cell sonicates and cellular fractionation

Since *P. gingivalis* produces a large amount of proteases, culture cells were suspended in a buffer (designated iTris) consisting of 20 mM Tris, pH 7.5, and three protease inhibitors; 10 mM phenylmethylsulfonyl fluoride, 1 mM N-α-tosyl-_L_-lysine chloromethyl ketone, and 1 mM leupeptin. The cells were disrupted by sonication, and whole-cell sonicates were obtained after remaining undisrupted cells were removed by centrifugation at 1,000×g for 10 min. The whole-cell sonicates were subjected to cellular fractionation as previously described [29]. Briefly, soluble and envelope fractions were separated by centrifugation at 100,000×g for 60 min. The envelope fraction was suspended in iTris supplemented with 1% Triton X-100 and 20 mM MgCl_2_, and then separated into soluble (inner membrane) and insoluble (outer membrane) fractions by centrifugation at 100,000×g for 60 min.

### Purification of FimA fimbriae

FimA fimbriae were purified from the wild-type 33277 and complemented strains as described previously [30]. Briefly, bacterial cells were gently suspended in 50 mM Tris, pH 7.5, supplemented with 150 mM NaCl and 10 mM MgCl_2_ by pipetting to release fimbriae from the cell surface without cell lysis. After the cells were removed by centrifugation, fimbriae were precipitated in 50% saturated ammonium sulfate. Then the fimbriae were further purified by DEAE Sepharose Fast Flow chromatography (GE Healthcare Bio-Sciences AB, Uppsala, Sweden). Purity was confirmed by SDS-PAGE and Coomassie Brilliant Blue (CBB) staining, and identity was confirmed by mass spectrometry as described previously [31]. For macrophage stimulation assay, we used the fimbrial samples after passage through a polymyxin B column, using Detoxi-Gel Endotoxin Removing Gel (Thermo Fisher Scientific Inc., Rockford, IL) to remove possible lipopolysaccharide (LPS) contamination. The lack of contamination by LPS was verified using Limulus ES-II Test Wako (Wako, Osaka, Japan).

### Animal experiments

All animal experiments were approved by the Aichi Gakuin University Animal Research Committee (permit number: AGUD120), and performed according to Regulations on Animal Experimentation at the University.

### Preparation of anti-FimX antisera

A *fimX* DNA fragment encoding FimX was amplified by PCR from 33277 chromosomal DNA. Primers used for construction are shown in Table S5. The DNA fragment was cloned into pET28(b) plasmid (Novagen Darmstadt, Germany), expressed in *Escherichia coli* BL21(DE3) with a N-terminal His tag. The cloned *fimX* was confirmed not to have unintended nucleotide alteration by DNA sequencing. For some unknown reasons, we could not purify His-tagged FimX with a Ni-affinity column; therefore we extracted His-tagged FimX from SDS-PAGE gel. The extracted protein was confirmed to be FimX by mass spectrometry. Anti-FimX antiserum was obtained by immunizing mice with extracted FimX emulsified with complete Freund's adjuvant.

### Immunization of mice with purified FimA fimbriae

SPF, female, 9-week old ICR mice (Chubu Kagaku Shizai Co., Ltd., Nagoya, Japan) were subcutaneously inoculated with purified FimA fimbriae emulsified with complete Freund's adjuvant. Six mice were used for each group. We checked the specific antibody titer after two inoculations at 2-week intervals, and booster injection was carried out again when the titer was low. Since some sera reacted to bacterial components of *P. gingivalis* other than FimA, we absorbed all antisera with FimA-deficient mutant cells of the 33277 Δ*mfa1* Δ*fim* cluster to reduce nonspecific reactions. The absorbed antisera were used for all experiments except for those presented in the supplemental material (Figs. S6 and S7).

### ELISA against whole-cell sonicates and purified FimA fimbriae

Whole-cell sonicates were prepared from the wild-type strains of *P. gingivalis*, and purified fimbriae were prepared from complemented strains as described above. Whole-cell sonicates at 50 µg/well or purified FimA fimbriae at 150 ng/well were coated on 96-well MaxiSorp Nunc-Immuno Plates (Thermo Fisher Scientific Inc.). After washing with 20 mM Tris, pH 7.5, supplemented with 150 mM NaCl and 0.05% Tween 20 (TBST), the wells were blocked with TBST supplemented with 5% bovine serum albumin. Next 1,000-fold diluted mouse sera were incubated. After the wells were washed and incubated with polyclonal rabbit anti-mouse immunoglobulins/HRP (Dako, Glostrup, Denmark), *o*-phenylenediamine and H_2_O_2_ in a citrate buffer, pH 5.0 was added as a substrate. The reaction was terminated with 1 M H_2_SO_4_, optical density at 490 nm (reference at 620 nm) was measured, and the values were used as antibody titers.

### Immunoblot analysis

Immunoblot analysis was performed by standard methods. Whole-cell sonicates and purified fimbriae were denatured by mixing with 5-fold concentrated loading buffer (1 M Tris, pH 6.8, 4% SDS, 50% glycerol, 20% 2-mercaptoethanol and bromophenol blue) and heating for 10 min, and then subjected to SDS-PAGE. We used specific antisera against monomeric and polymeric FimA [30], PgmA [13], and FimX as described above. We also used antisera from mice immunized with purified FimA fimbriae as described above. ECL prime (GE Healthcare Bio-Sciences AB) with high sensitivity was used for the detection.

### Transmission electron microscopy

Bacterial cells and purified FimA fimbriae were negatively stained with 1% ammonium molybdate, and observed with a JEM-1210 transmission electron microscope (TEM) (JEOL Ltd., Tokyo, Japan).

### Macrophage stimulation assay

Mouse macrophage-like J774-1cells were provided by the RIKEN BRC through the National Bio-Resource Project of the MEXT, Japan, and maintained in RPMI 1640 (Cat# R8758, Sigma-Aldrich, St. Louis, MO) supplemented with 10% heat-inactivated fetal bovine serum, 100 U/ml penicillin and 100 µg/ml streptomycin at 37°C under 5% CO_2_. J774-1 cells were seeded at 2×10^5^ cells/well in a 48-well plate, and incubated for 2 days. The medium in each well was replaced with RPMI medium containing LPS-free FimA fimbriae at 1 µg/ml or LPS at 10 EU/ml (corresponding to 2.6 ng/ml of LPS from *E. coli* UKT-B, Wako), then incubated for 3 to 24 hours. It was estimated that 1 µg of FimA fimbriae corresponded to 10^9^ cells in the case of 33277. J774-1 cells reached nearly confluent status after 24-hour incubation. TNF-α secreted into the medium was measured using Mouse TNF alpha ELISA Ready-SET-Go! (eBioscience, San Diego, CA).

### Statistics

Statistical results are expressed as means ± standard deviations (SD). One-way analysis of variance and the Dunnett multiple-comparison test were used to evaluate differences between groups. Significance was defined as a *p* value of <0.05.

## Results

### Complementation of fimX, pgmA and fimA to fim cluster-deletion mutant

**Figure 2:**
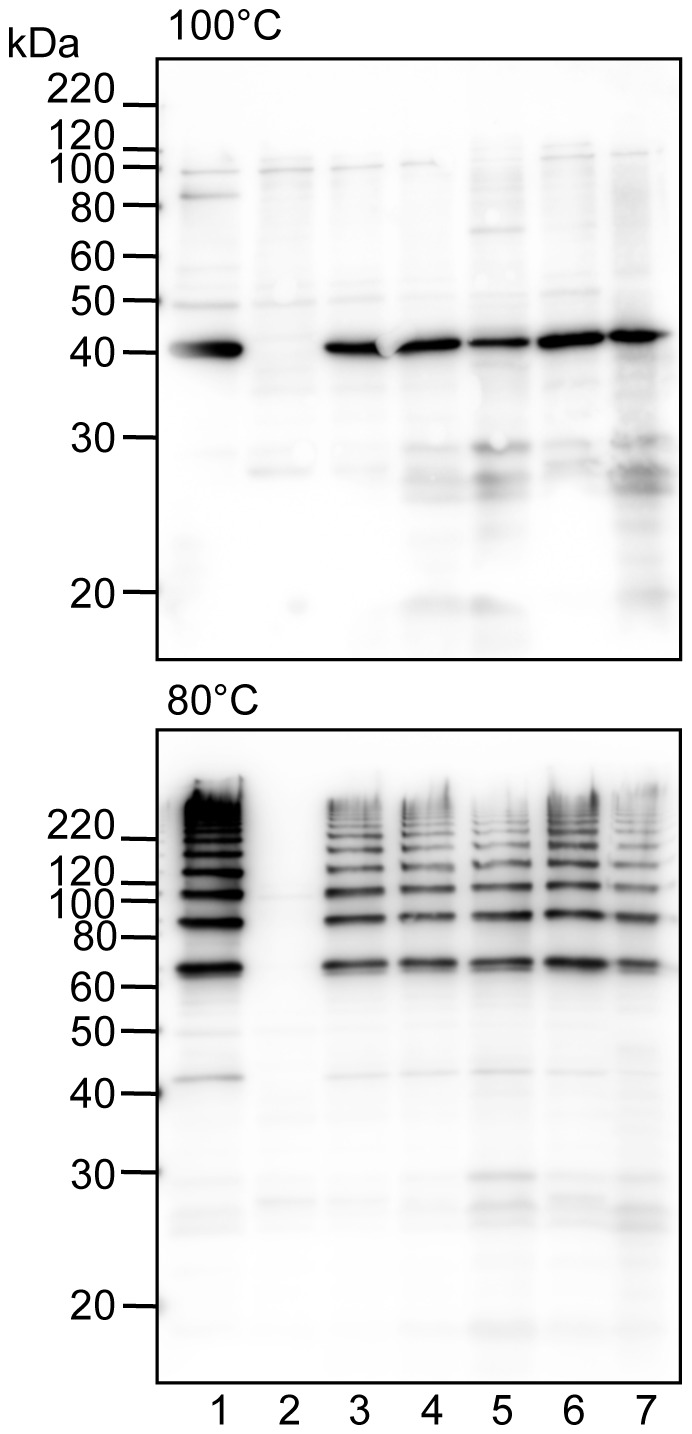
Immunoblot analysis for FimA using whole-cell sonicates. Whole-cell sonicates were denatured in an SDS-containing buffer with 2-mercaptoethanol by heating at 100°C (upper) and 80°C (lower) for 10 min, then subjected to SDS-PAGE and immunoblot analysis. A mixture of specific antisera to the FimA polymer and monomer was used. Antigen samples were as follows: *P. gingivalis* ATCC 33277 Δ*mfa1* (expresses native FimA fimbriae, lane 1), *P. gingivalis* ATCC 33277 Δ*mfa1* Δ*fim* cluster (FimA deficient, lane 2) with *fimX-pgmA-fimA* (lane 3), *pgmA-fimA* (lane 4), *fimA* (lane 5), *fimX* & *fimA* (lane 6) complementarily introduced, and *P. gingivalis* W83 Δ*fim* cluster with *fimA* introduced (lane 7). All introduced genes originated from *P. gingivalis* ATCC 33277. Incomplete dissociation of FimA polymers produces a ladder-like band indicating oligomers in the lower panel. Note that bands slightly higher than 60 kDa (the lower panel) are dimers although monomers appear to be about 40 kDa (the upper panel).

**Figure 3:**
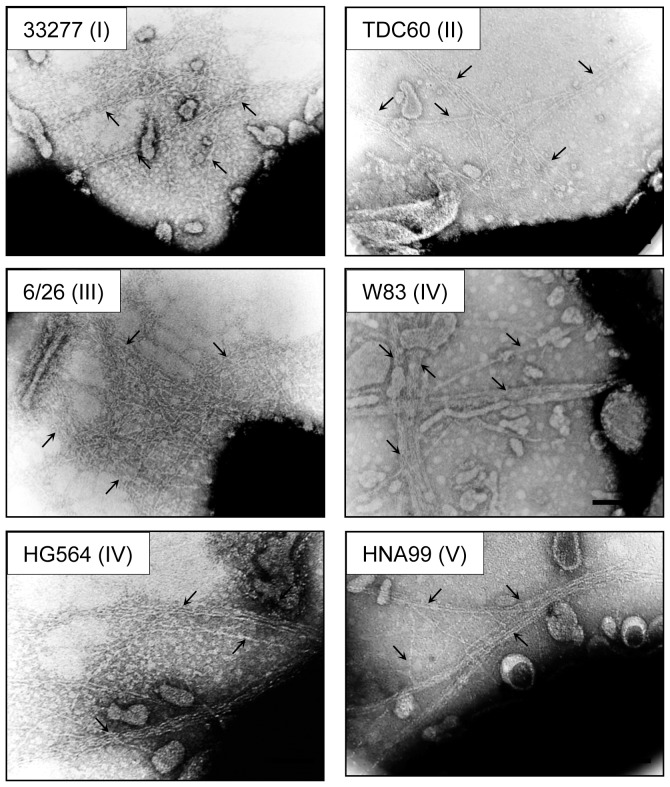
Transmission electron microscopic observation of FimA fimbriae on the bacterial cell surface. *P. gingivalis* ATCC 33277 Δ*mfa1* Δ*fim* cluster cells with *fimA* from 33277, TDC60, 6/26, W83, HG564 and HNA99 introduced by using an expression vector. Samples were negatively stained with 1% ammonium molybdate. Arrows indicate fimbrial structure. Some fimbriae appear to be bundled. Bars show 0.2 µm.

It is reported that the loss of *fimX* and *pgmA* abolishes or decreases FimA protein production [12], but it is still unclear whether they play a vital role in polymerization of FimA. We first examined their roles in FimA polymerization using a complementation system. We cloned and constructed DNA fragments of *fimX-pgmA-fimA*, *pgmA-fimA*, *fimX* & *fimA*, and *fimA* from 33277, and then complementarily introduced them into the whole *fim* cluster-deletion mutant 33277 Δ*mfa1* Δ*fim* cluster through an expression vector. FimA expression and polymerization were examined by SDS-PAGE and immunoblot analysis by using a mixture of specific antisera to monomeric and polymeric FimA. The FimA polymer was mostly dissociated to monomers in SDS buffer by heating at 100°C. In contrast, when it was heated at a lower temperature such as at 80°C, the dissociation of polymers only partially occurred, and ladder-like bands indicating oligomers were observed [32]. Although we examined all combinations of *fimA* with *fimX* and *pgmA*, the *fimA* gene alone was able to express the FimA protein, and was sufficient to form the oligomers (Fig. 2, lanes 5). There was no obvious alteration in bands caused by introduction of *fimX* and *pgmA* (Fig. 2, lanes 3, 4 and 6). Additionally, even when the W83 Δ*fim* cluster was used as the host, 33277 *fimA* solely conferred fimbrial expression (Fig. 2, lane 7). TEM observation confirmed that the cells carrying *fimA* as the sole gene in the *fim* cluster expressed long FimA fimbriae (Fig. 3, panel of 33277).

**Figure 4:**
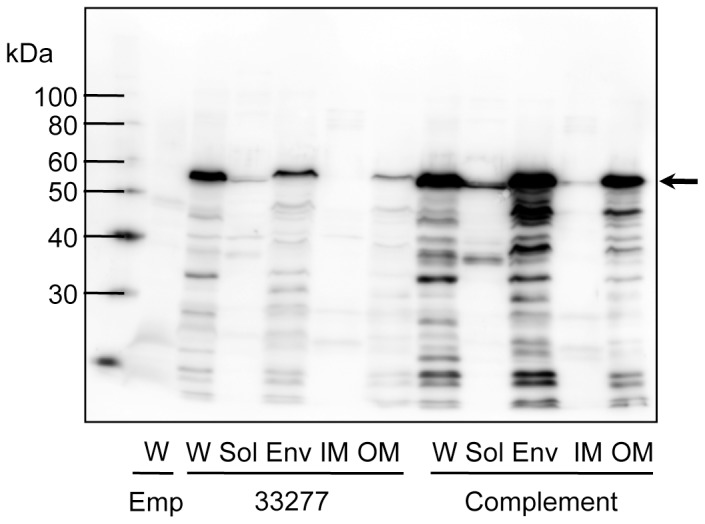
Immunoblot analysis for PgmA using whole-cell sonicates. Whole-cell sonicates (W) were fractionated into soluble (Sol), envelope (Env), inner membrane (IM) and outer membrane (OM) fractions. Samples were denatured in an SDS-containing buffer with 2-mercaptoethanol by heating at 100°C for 10 min, then subjected to SDS-PAGE and immunoblot analysis. Emp denotes 33277 Δ*mfa1* Δ*fim* cluster/pT-COW::*ragAP*, carrying empty vector, used as a negative control; 33277 denotes the wild-type strain; Complement denotes 33277 Δ*mfa1* Δ*fim* cluster carrying pT-COW::*ragAP*::*fimX-pgmA-fimA*. An arrow indicates PgmA as a 60-kDa protein. Degradation bands (below the 60-kDa) were also visualized because PgmA was highly sensitive to intrinsic proteases of this bacterium [13].

We examined whether FimX and PgmA were localized in an appropriate site in the complemented cells. PgmA was detected in the outer membrane fraction in the complement as well as in the wild-type strain (Fig. 4), whereas FimX was not detected in cells of the complement or the wild-type strain (data not shown).

Taken together, these findings indicated that, within the *fim* cluster, *fimA* alone conferred the ability to express long FimA fimbriae, and the other genes were not essential for polymerization of FimA.

### Introduction of fimA from each genotype into the fim cluster-deletion mutant

*fimA* genes including types I–V were introduced into the 33277 Δ*mfa1* Δ*fim* cluster using an expression vector. With regard to type IV, since it was reported that FimA protein was not polymerized into fimbriae in W83 even when it was forcedly produced in the cell through gene manipulation [25], another type IV strain HG564 was also examined. After it was subcloned in *E. coli*, the cloned *fimA* DNA was sequenced. With respect to genome-sequenced strains of 33277, TDC60 and W83, sequences of the corresponding cloned genes were in agreement, though there were some differences from the data deposited in the databank for 6/26, HG564 and HNA99. However, the cloned DNA sequences were completely identical to the sequences of chromosomal DNA analyzed by us (Supporting Information S1).

**Figure 5:**
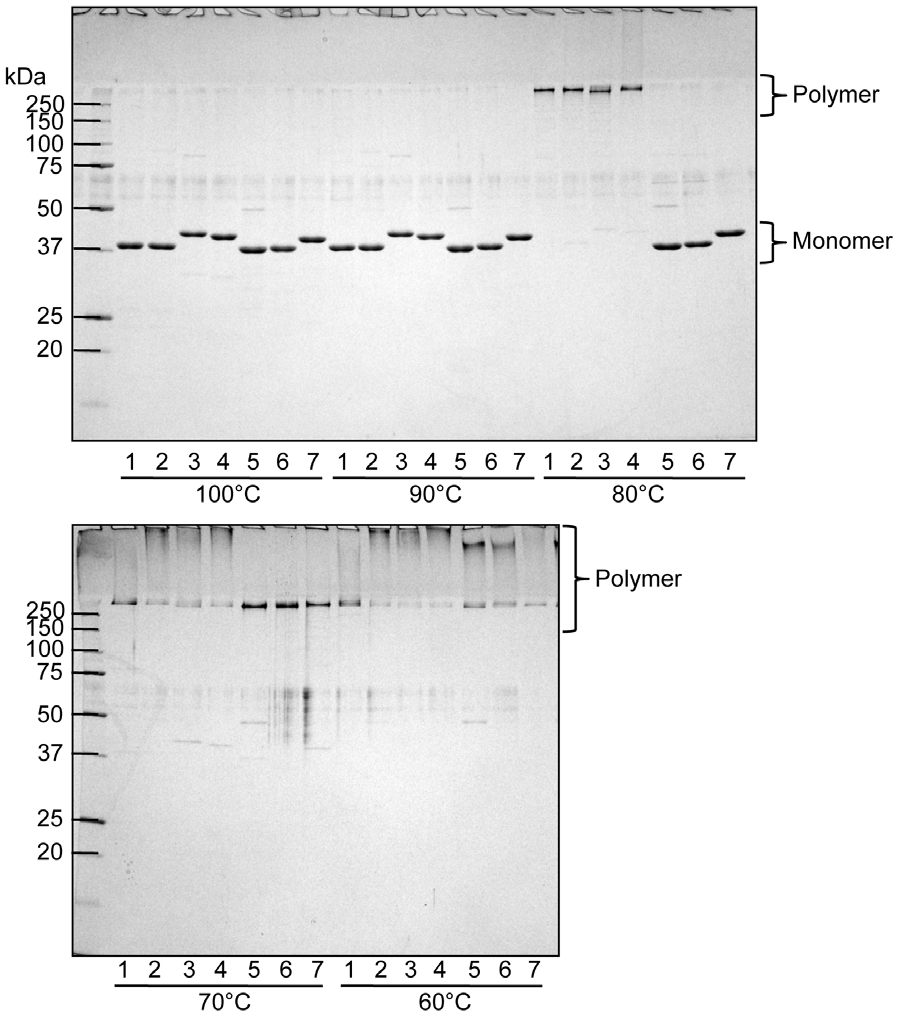
SDS-PAGE and CBB staining using purified FimA fimbriae. Purified FimA fimbriae were denatured in an SDS-containing buffer with 2-mercaptoethanol by heating at 60 to 100°C for 10 min, then subjected to SDS-PAGE and CBB staining. Samples were as follows: purified from *P. gingivalis* ATCC 33277 Δ*mfa1* (native 33277 FimA fimbriae, lane 1), *P. gingivalis* ATCC 33277 Δ*mfa1* Δ*fim* cluster with *fimA* of ATCC 33277 (I) (lane 2), TDC60 (II) (lane 3), 6/26 (III) (lane 4), W83 (IV) (lane 5), HG564 (IV) (lane 6), and HNA99 (V) (lane 7) introduced. Note that CBB staining did not visualize a ladder band as seen in immunoblot analysis in Fig. 2.

As shown in Fig. 3, all genotypes expressed long fimbrial structures. In contrast to a previous report [25], a strain complemented with W83 *fimA* expressed a fimbrial structure. However, all complements produced many vesicles, indicating that they were in stressful condition. Therefore it was difficult for us to obtain fine images of fimbriae on the surface. We then purified fimbriae derived from each genotype (Fig. S5) and subjected them to further analyses. Purified fimbriae were denatured at various heating temperatures, and subjected to SDS-PAGE analysis with CBB staining (Fig. 5). In addition to native 33277 FimA fimbriae, the polymeric structures of fimbriae types I, II and III were substantially maintained at 80°C, although their polymers were mostly dissociated into monomers at 90 and 100°C. On the other hand, fimbriae of types IV and V dissociated to monomers at 80°C although they appeared to be intact at 70°C and under.

### Reactivity of anti-FimA fimbriae antiserum of each genotype

**Figure 6:**
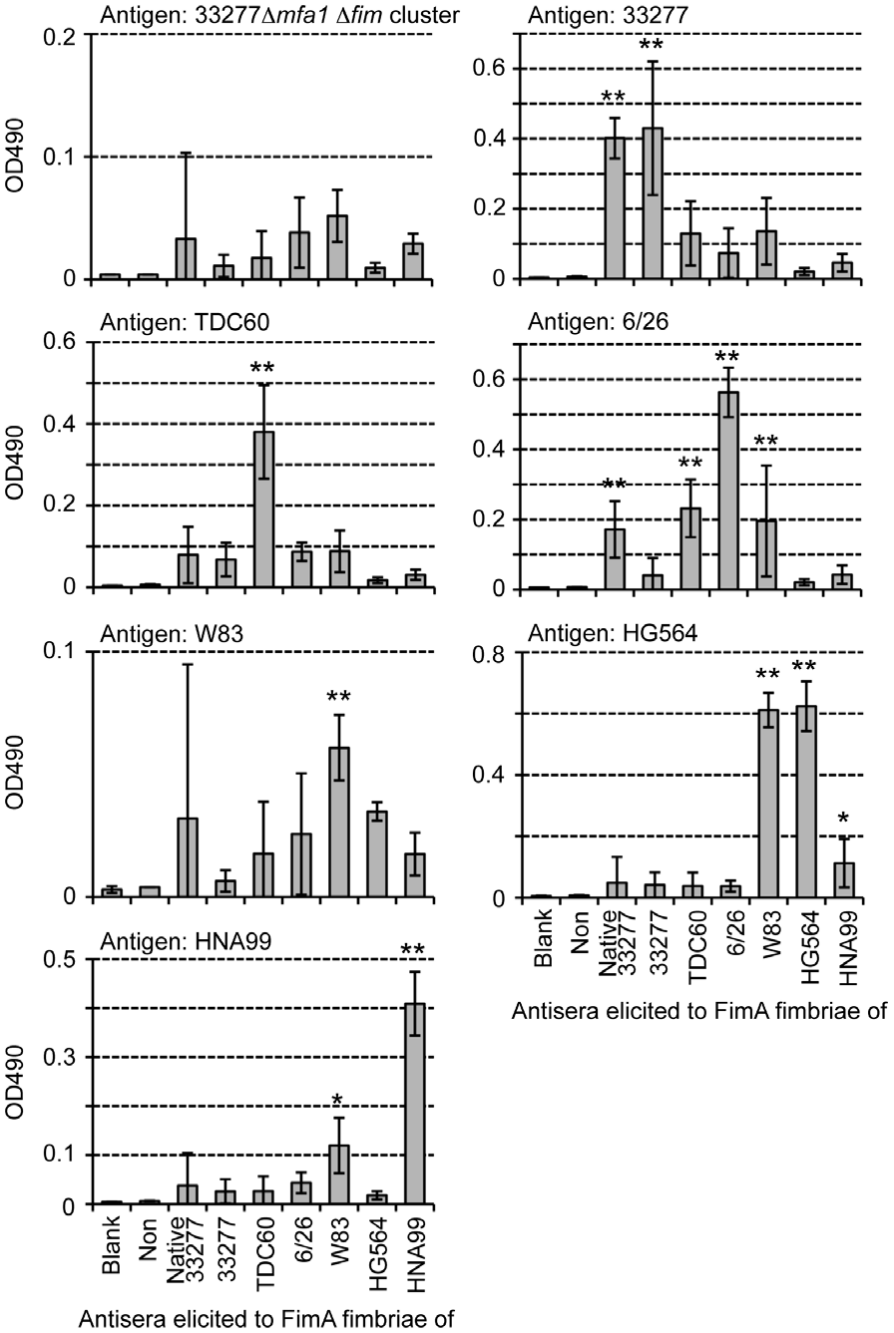
ELISA using absorbed antisera and whole-cell sonicates as antigen. Whole-cell sonicates were coated on ELISA plates as antigens. Antisera from mice immunized with each pure genotype fimbriae were used after absorption with the fimbria-deficient mutant 33277 Δ*mfa1* Δ*fim* cluster. “Non” indicates non-immunized mouse sera. W83 rarely produces FimA protein and fimbriae. Data show mean ± SD. Asterisks indicate statistical significance compared with Non (* *p*<0.05, ** *p*<0.01). Note that scales of Y axes are adjusted in order to compare titers clearly.

**Figure 7:**
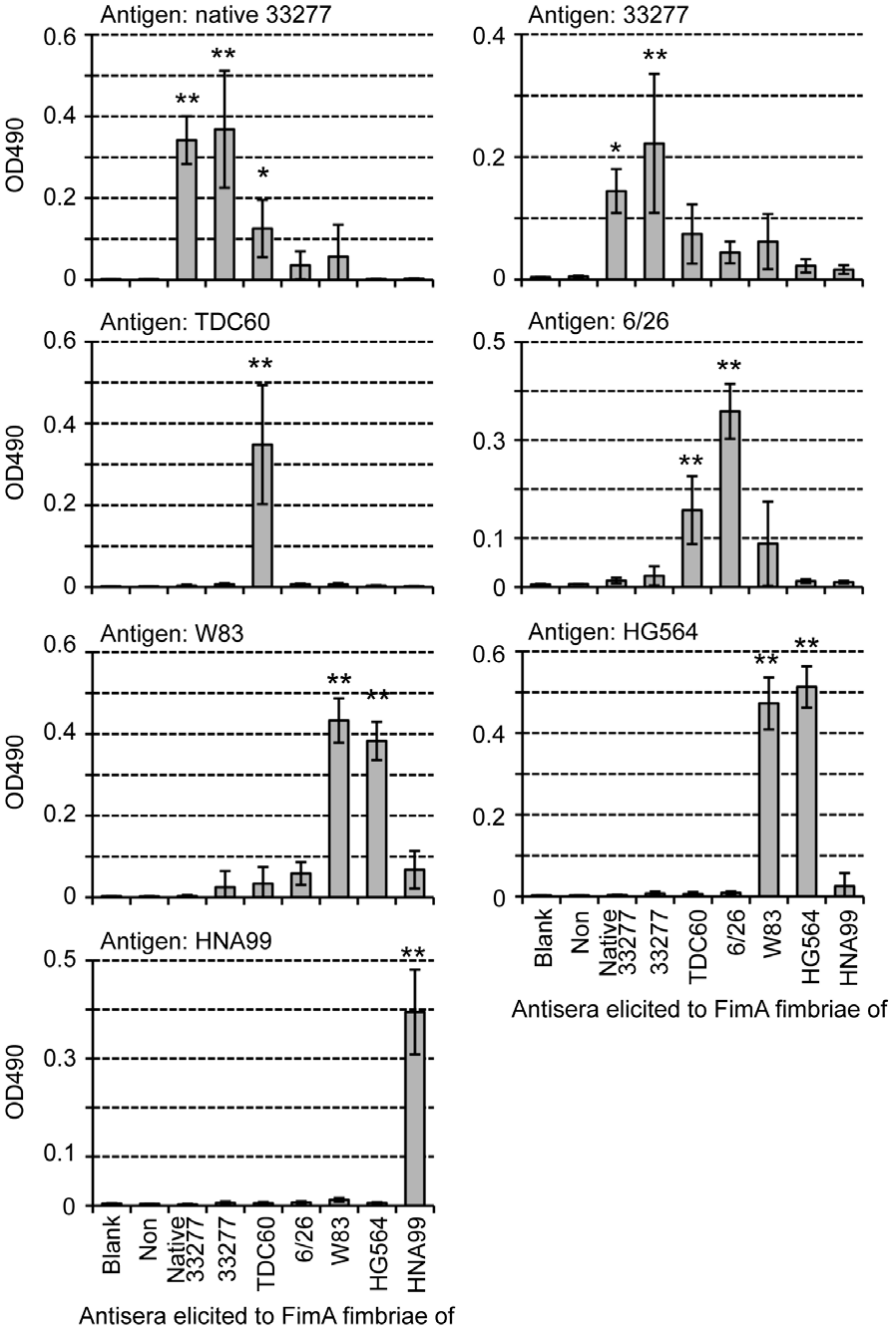
ELISA using absorbed antisera and purified FimA fimbriae as antigen. Pure FimA fimbriae, derived from each *fimA* gene, were coated on ELISA plates as antigens. Antisera from mice immunized with each pure genotype fimbriae were used after absorption with the fimbria-deficient mutant 33277 Δ*mfa1* Δ*fim* cluster. “Non” indicates non-immunized mouse sera. Data show mean ± SD. Asterisks indicate statistical significance compared with Non (* *p*<0.05, ** *p*<0.01). Note that scales of Y axes are adjusted as in Fig. 6.

Antisera against all genotypes of FimA fimbriae were successfully obtained. No protein contaminants appeared in the purified fimbrial samples, as shown in Fig. 5. However, ELISA using whole-cell sonicates as antigens showed that some antisera reacted to bacterial components, presumably including LPS, other than FimA (Fig. S6). Thus, we absorbed antisera with cells of the FimA-deficient strain 33277 Δ*mfa1* Δ*fim* cluster, and used them for ELISA in Fig. 6. Although ELISA using purified fimbriae showed fairly antigen-specific reactions even when we used sera without absorption (Fig. S7), we preferred to use absorbed antisera as shown in Fig. 7.

Fig. 6 shows results of ELISA using whole-cell sonicates of the wild-type strains. All antibody titers against fimbria-deficient mutants of the 33277 Δ*mfa1* Δ*fim* cluster were decreased to the background level by the absorption. Antisera of mice immunized with native 33277 FimA fimbriae (anti-native 33277 antisera) and 33277 FimA fimbriae (anti-33277 antisera) reasonably showed high titers in response to 33277 cells, whereas other antisera reacted much less against them. Similarly, anti-TDC60 antisera alone showed a high titer to TDC60 cells. Against 6/26 cells, anti-6/26 antisera showed the highest titer, but some antisera also showed moderate cross-reactivity. Because W83 rarely produces FimA protein and fimbriae, all antisera showed a titer at background level. Anti-W83 and anti-HG564 antisera showed high titers to HG564 cells, whereas anti-HNA99 antisera showed the highest titer to HNA99 cells. However, anti-W83 and anti-HNA99 antisera slightly cross-reacted with HNA99 and HG564 cells, respectively. These results indicated that antisera elicited by purified FimA fimbriae were generally genotype specific, although minor cross-reactivity was observed. ELISA using purified fimbriae reinforced the finding that they were clearly genotype-specific (Fig. 7). Both anti-native 33277 and anti-33277 antisera showed high titers to native 33277 FimA fimbriae, and they had a high correlation coefficient (*r* = 0.68). Similarly, these antisera reacted well to 33277 FimA fimbriae, with high correlation (*r* = 0.87). Anti-TDC60 antisera solely recognized TDC60 FimA fimbriae. Against 6/26 FimA fimbriae, anti-6/26 antisera showed the highest titer, and anti-TDC60 and anti-W83 antisera tended to cross-react. Anti-W83 and anti-HG564 antisera reacted to both W83 and HG564 FimA fimbriae (both genotype IV), and had a high correlation coefficient (*r*>0.86). Anti-HNA99 antisera had a high titer only to HNA99 FimA fimbriae.

**Figure 8:**
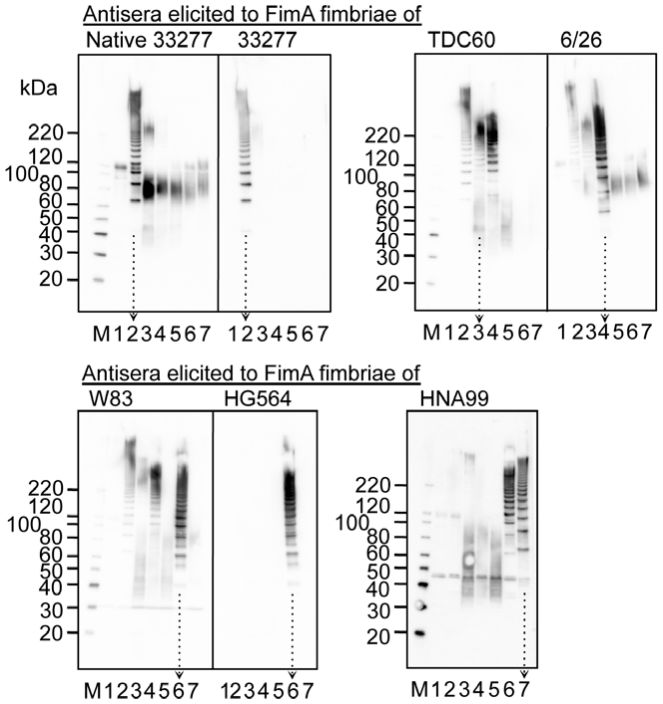
Immunoblot analysis using whole-cell sonicates partially denatured. Whole-cell sonicates were denatured in an SDS-containing buffer with 2-mercaptoethanol by heating at 70°C for 10 min, and subjected to SDS-PAGE and immunoblot analysis by using antisera, 1,000-fold dilution, from mice immunized with purified FimA fimbriae. Antigen samples were as follows: *P. gingivalis* ATCC 33277 Δ*mfa1* Δ*fim* cluster (FimA deficient, lane 1), and the wild-type strains of ATCC 33277 (lane 2), TDC60 (lane 3), 6/26 (lane 4), W83 (lane 5), HG564 (lane 6), and HNA99 (lane 7). M denotes a standard marker. W83 rarely produces FimA protein and fimbriae. Note that ladder bands are specific for FimA fimbriae whereas smear bands between 40–80 kDa are nonspecific. Arrows with dotted lines are placed in order to clearly discriminate each lane.

**Figure 9:**
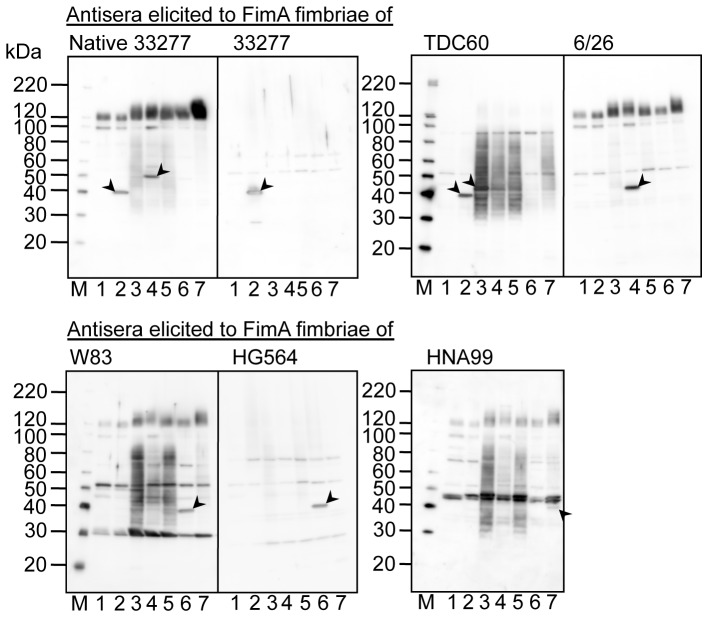
Immunoblot analysis using whole-cell sonicates completely denatured. Whole-cell sonicates were denatured in an SDS-containing buffer with 2-mercaptoethanol by heating at 100°C for 10 min, and subjected to SDS-PAGE and immunoblot analysis using antisera, 1,000-fold dilution, from mice immunized with purified FimA fimbriae. Antigen samples were as follows: *P. gingivalis* ATCC 33277 Δ*mfa1* Δ*fim* cluster (FimA deficient, lane 1), and the wild-type strains of ATCC 33277 (lane 2), TDC60 (lane 3), 6/26 (lane 4), W83 (lane 5), HG564 (lane 6), and HNA99 (lane 7). M denotes standard marker. Arrowheads show distinguishable bands corresponding to FimA monomers. Note that W83 rarely produces FimA protein and fimbriae.

We also conducted immunoblot analysis (Figs. 8 and 9). Whole-cell sonicates of the wild-type strains were denatured by heating at 70 and 100°C as shown in Figs. 8 and 9, respectively, then subjected to SDS-PAGE. Antisera elicited with the same antigen were pooled and used for immunoblot analyses. Fig. 8 shows antigen-specific reactivity to partially dissociated FimA polymers as a ladder-like band. All antisera showed ladder-like bands in response to cell sonicates containing corresponding FimA fimbriae. However, some antisera showed ladder-like bands in response to different genotypes. Anti-TDC60 and anti-W83 antisera also reacted to 33277 and 6/26, anti-HNA99 antisera reacted to HG564, and anti-6/26 antisera reacted marginally to 33277 and TDC60. In addition, antisera other than anti-33277 and anti-HG564 antisera showed nonspecific bands as smear bands, although nonspecific reactions were reduced due to absorption. Fig. 9 shows reactivity to monomeric FimA. Intensities of bands corresponding to the FimA monomer were very low in all reactions. Although reactivities to FimA monomers were weak, the reactions were genotype specific, except that anti-native 33277 antisera also exhibited a band against 6/26, and anti-TDC60 antisera did so against 33277.

### Induction of a proinflammatory cytokine in macrophages by FimA fimbriae

**Figure 10:**
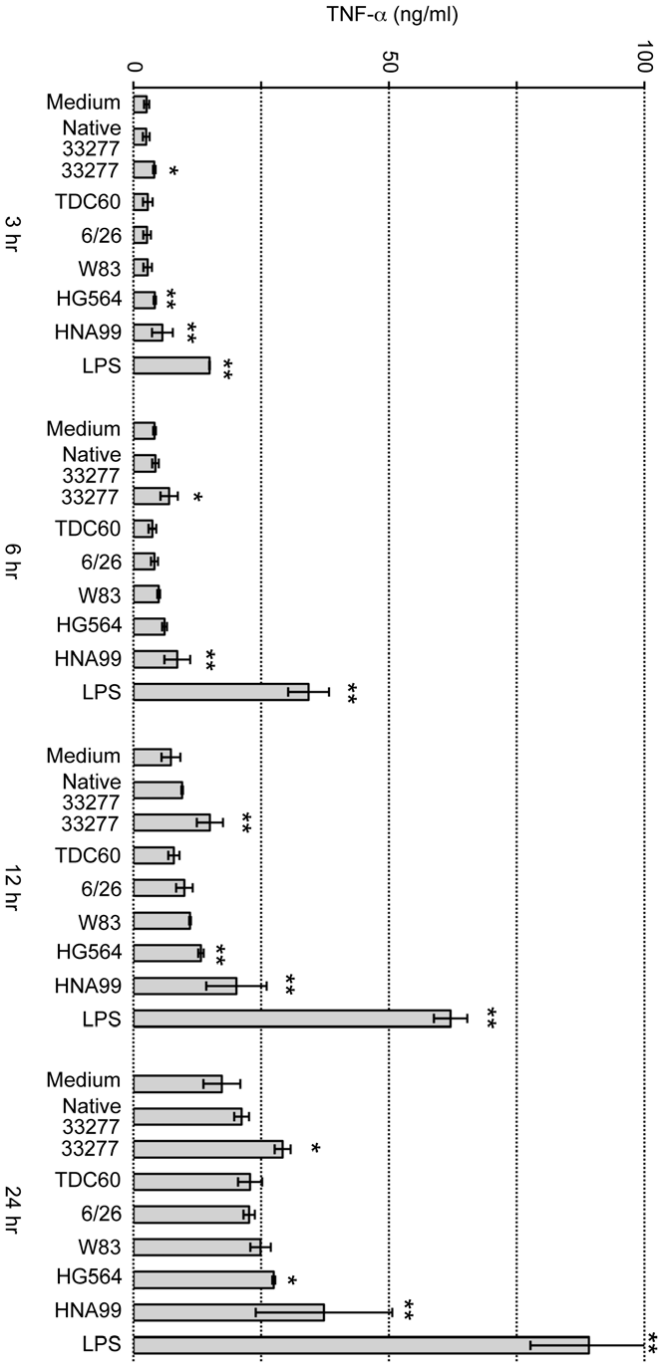
TNF-α induction in mouse macrophage-like J774-1 cells. Purified FimA fimbriae at 1 µg/ml and LPS at 10 EU/ml (corresponding to 2.6 ng/ml) were incubated with J774-1 cells for 3 to 24 hours. TNF-α in the medium was measured by ELISA. Medium denotes no addition; Native 33277 denotes purified FimA fimbriae from 33277 Δ*mfa1*; 33277, TDC60, 6/26, W83, HG564 and HNA99 denote that pure FimA fimbriae, derived from the corresponding *fimA* in *P. gingivalis* ATCC 33277 Δ*mfa1* Δ*fim* cluster, were used stimulants; LPS was *E. coli* LPS. Data show mean ± SD from two experiments with duplicate. Asterisks indicate statistical significance compared with Medium for each time (* *p*<0.05, ** *p*<0.01).

It is known that LPS strongly stimulates macrophages and induces proinflammatory cytokines such as TNF-α. As described above, LPS possibly contaminated the purified FimA fimbrial samples. Therefore, the fimbrial samples were passed through a polymyxin B column to remove LPS. Macrophage-like cell line J774-1 was incubated with purified FimA fimbriae or *E. coli* LPS as a positive control, and samples were sequentially collected to measure the TNF-α concentration (Fig. 10). TNF-α gradually increased even without any addition to the medium, and *E. coli* LPS strongly elicited TNF-α each time. No native or recombinant fimbriae (genotypes I to V) showed strong activity as compared with *E. coli* LPS, although fimbriae of some genotypes showed slightly higher values than in the “medium” used as a negative control.

## Discussion

By complementary introduction of *fimA* alone into the whole *fim* cluster-deletion mutants (constructed from 33277 and W83), the complemented strains expressed FimA fimbriae with substantial length (Figs. 2 and 3). It was reported that genetic inactivation of *fimX* and *pgmA* resulted in drastic reduction in *fimA* transcription due to malfunction of the two-component regulatory system [12]. However, we used an expression vector to produce FimA protein independently of the system, and demonstrated that neither *fimX* or *pgmA* was essential for the fimbrial assembly. Additionally, it was not observed that introductions of *fimX* and *pgmA* promoted fimbriation. PgmA was localized in the outer membrane even in the complement (Fig. 4). However, FimX was not detected. Although it is unclear why it was not detected, its half-life might be very short, or the anti-FimX antisera could not recognize native FimX because His-tagged FimX of immunogen was prepared as denatured form described in Materials and Methods. Therefore, we could not obtain further information about properties of PgmA and FimX in this study. Our results also clearly showed that, by using a *fim* cluster-deletion mutants, the accessory components FimCDE were not essential for polymerization and elongation. These minor components were expected to be necessary for polymerization/elongation, because deficiency of FimC, FimD, or FimE caused a decrease of the fimbrial expression [10], [11], [33]. Our results suggest that the decrease of the fimbrial expression resulted from a decrease of production of FimA protein, but not from a decrease of polymerization/elongation efficiency. Indeed, deletion of *fimCDE* resulted in a decrease of production of FimA protein (unpublished data). Shoji *et al.* reported that it was essential for FimA to undergo processing by proteases of this bacterium and lipidation for the fimbrial assembly [33], [34]. Thus, our results also showed that genes in the *fim* cluster were not critically involved in the processing. It seems that *P. gingivalis* has a quite unique mechanism for FimA fimbrial formation, and further studies are required.

Since *fimA* alone conferred the fimbrial formation, we next transformed the *fim* cluster-deletion mutant with *fimA* cloned from each genotype strain, including types I to V. TEM observation showed that all genotypes of *fimA* could express long FimA fimbriae (Figs. 3 and S5). In addition, the high molecular weight polymer bands shown in Fig. 5 indicate that FimA was polymerized, supporting the finding that all genotypes formed fimbriae. There have been reports that FimA fimbriae appear to be morphologically different between genotypes, especially the fimbrial length; type I strains had long fimbriae, whereas type II and IV strains were short [15], [16]. Since the often-used *P. gingivalis* strains 33277 and 381, both are type I, express long fimbriae several micrometers in length, it has been believed that type I produces long fimbriae. But we found that they have a deficiency of FimB by a nonsense mutation in *fimB*, and FimB restoration in 33277 resulted in production of short FimA fimbriae, about 150 nm in length [8]. Additionally, we showed previously that FimB functioned as a terminator of FimA fimbriae, and the length of the fimbriae was regulated by the expression ratio of FimA and FimB [8]. In the present study, since we used a FimB-deficient host strain, the fimbriae were likely to become long in all genotypes. We would like to emphasize that FimA itself does not have a property to regulate the length. However, fimbriae of types I, II and III were slightly more thermostable in SDS buffer than those of types IV and V (Fig. 5), suggesting that there may be biological differences in the fimbriae of the various genotypes.

TEM showed excessive vesicle formation in the complemented strains. Deletion of whole *fim* cluster tended to render the cell surface unstable. Since PgmA and FimB in the cluster are the outer membrane proteins, they could contribute to stabilize the surface, especially, the outer membrane.

The results of ELISA and immunoblot analyses are summarized in Table 1. Although they were not completely monospecific, the antisera mostly showed genotype-specific reactivity, indicating that there was a relationship between the genotype and serotype. One of us has already reported that there are serotypes in FimA fimbriae [35]; agglutination, Ouchterlony and immunoblot analyses showed that some *P. gingivalis* strains did not react with an antiserum to type I FimA fimbriae purified from strain 381. Lee *et al.* [36] also examined reactivity against fimbriae extracted from various *P. gingivalis* strains by using an antiserum elicited with type I FimA fimbriae, and they reported that FimA fimbriae had antigenic heterogeneity. In our study, we systematically prepared antisera against FimA fimbriae of five genotypes, evaluated the antigenicity quantitatively and qualitatively using ELISA and immunoblotting, and demonstrated that there was differential antigenicity among the genotypes. It is thus necessary to further investigate serotypes using clinical isolates.

**Table 1:** Summary of serological analyses.

Antisera against	Experiment*		Antigen					
			33277**	TDC60	6/26	W83	HG564	HNA99
Native 33277 (I)	ELISA	Cell	+++	−	++	−	−	−
		Pure	+++	−	−	−	−	−
	IB	Poly	++	−	−	−	−	−
		Mono	+	−	+	−	−	−
33277 (I)	ELISA	Cell	+++	−	−	−	−	−
		Pure	+++	−	−	−	−	−
	IB	Poly	+++	−	−	−	−	−
		Mono	+	−	−	−	−	−
TDC60 (II)	ELISA	Cell	+	+++	++	−	−	−
		Pure	++	+++	++	−	−	−
	IB	Poly	++	+	++	−	−	−
		Mono	+	+	+	−	−	−
6/26 (III)	ELISA	Cell	+	−	+++	−	−	−
		Pure	−	−	+++	−	−	−
	IB	Poly	+	+	+++	−	−	−
		Mono	−	−	+	−	−	−
W83*** (IV)	ELISA	Cell	−	−	++	−	+++	++
		Pure	−	−	+	+++	+++	−
	IB	Poly	+	−	+	−	++	−
		Mono	−	−	−	−	+	−
HG564 (IV)	ELISA	Cell	−	−	−	−	+++	−
		Pure	−	−	−	+++	+++	−
	IB	Poly	−	−	−	−	+++	−
		Mono	−	−	−	−	+	−
HNA99 (V)	ELISA	Cell	−	−	−	−	+	+++
		Pure	−	−	−	−	−	+++
	IB	Poly	−	−	−	−	++	++
		Mono	−	−	−	−	−	+

* Experiment; ELISA/Cell, ELISA/Pure, IB/Poly and IB/Mono are from Figs. 6, 7, 8, and 9, respectively.

** Antigen of 33277 in ELISA/Pure is 33277 FimA fimbriae, not native 33277.

*** W83 rarely produces FimA protein and fimbriae.

IB, immunoblot; Cell, whole-cell sonicates; Pure, purified FimA fimbriae; Poly, partially dissociated FimA fimbriae; Mono, FimA monomer.

+++ reacted strongly; ++ reacted moderately; + reacted weakly; − did not react.

There are partially common or similar amino acid sequences among genotypes. Types II and III are very similar over their full lengths (Information S2 and Fig. S8). Nevertheless, cross-reactivity was considerably low. We think one reason for this is that antibodies preferentially recognize a conformation or a discontinuous epitope of FimA polymers. Indeed, reactivities to monomers were very low as shown in Fig. 9. One of us also has already reported this. Anti-FimA polymer antiserum, elicited by the fimbriae (polymer), principally reacted to the polymeric structure but not to the monomer, whereas anti-monomer antiserum, elicited by fimbrilin (monomer), showed an opposite tendency [32], [35]. Ito *et al*. [37] also reported that they produced monoclonal antibodies from mice immunized with FimA fimbriae of *P. gingivalis*, and showed that all monoclonal clones did not recognize the monomer but rather the polymer of the fimbriae. In addition, the same probably applies to reactions in patients with periodontal diseases; the sera from patients had a strong tendency to react with polymer, but not with monomer [38]. It is likely that a specific antibody to FimA fimbriae is predominantly induced against the polymeric conformation, and the polymeric conformations have different antigenic determinant epitopes among genotypes.

As described above, even though polyclonal antibodies were used, FimA fimbriae are fairly genotype-specific as far as discontinuous epitopes are mainly recognized. However, since they contain common or similar primary sequences among genotypes as potential continuous epitopes, cross-reactive antibodies to the common epitopes could be produced depending on immunization conditions such as frequent immunizations or immune responses of animals as far as polyclonal antibodies are concerned.

Antisera elicited by purified fimbriae showed similar specific reactivities to both purified fimbriae from complemented strains and whole-cell sonicates of the wild-type strains. Purified fimbriae were practically derived from recombinant proteins because they were obtained from the *fim*-cluster deletion mutant carrying *fimA* alone, whereas whole-cell sonicates express native FimA fimbriae. These results indicated that “recombinant” FimA fimbriae produced by *fimA* alone showed the same conformation as native ones.

Finally, no genotype of FimA fimbriae showed strong stimulative activity for a macrophage cell line, even when fimbriae were added at 1 µg/ml, the amount corresponding to about 10^9^ cells/ml of *P. gingivalis*. Some studies using type I FimA fimbriae such as 33277 reported that the fimbriae induced proinflammatory cytokine production [39], [40], [41]. However, a more recent study demonstrated that highly purified type I FimA fimbriae did not cause such activity [42]. We showed here that other genotypes did not have obvious activity for cytokine induction either. Although we used native type I FimA fimbriae, with accompanying accessory components, other genotypes of fimbriae were solely composed of FimA. This should be confirmed by using native fimbriae since accessory components are thought to be potential immunomodulators [11].

In conclusion, we showed here that *fimA* alone expresses FimA fimbriae, and that antisera against FimA fimbriae have genotype-specific reactivity.

## Supporting Information

Information S1 DNA sequences analyzed in this study.
(DOC)

Information S2 Multiple sequence alignment between FimA fimbriae of *P. gingivalis* strains by ClustalW.
(DOC)

Figure S1 **Construction of a** ***mfa1-*** **deletion mutant of** ***P. gingivalis*** **.** Small arrows show the primers.
(PDF)

Figure S2 **Construction of a** ***fim*** **cluster-deletion mutant of** ***P. gingivalis*** **.** Small arrows show the primers. *ermF-ermB* confers erythromycin resistance to *P. gingivalis. ermB* was previously called *ermAM*, but the current nomenclature proposed to use *ermB* (Roberts *et al*., Antimicrob. Agents Chemother. 1999, 43: 2823–30), therefore we used here *ermB*.
(PDF)

Figure S3 **Complementary introductions of** ***fim*** **-cluster genes into the** ***fim*** **cluster-deletion mutant of** ***P. gingivalis*** **.** *fimX-pgmA-fimA*, *pgmA-fimA*, *fimX* & *fimA*, or *fimA* gene were introduced into *fim* cluster-deletion mutant of *P. gingivalis*. Small arrows show the primers. *tetQ* confers tetracycline resistance to *P. gingivalis*.
(PDF)

Figure S4 **Introduction** **of the** ***fimA*** **gene of each genotype into the** ***fim*** **cluster-deletion mutant of** ***P. gingivalis*** **.** The *fimA* gene from various *P. gingivalis* strains including ATCC 33277 (type I), TDC60 (type II), 6/26 (type III), W83 (type IV), HG564 (type IV), and HNA99 (typeV) were introduced into *fim* cluster-deletion mutant of *P. gingivalis*. Small arrows show the primers. *tetQ* confers tetracycline resistance to *P. gingivalis*.
(PDF)

Figure S5 **Transmission electron microscopic observation of FimA fimbriae purified from complements.** FimA fimbriae were purified from *P. gingivalis* ATCC 33277 Δ*mfa1* (Native 33277), and *P. gingivalis* ATCC 33277 Δ*mfa1* Δ*fim* cluster cells with *fimA* from 33277, TDC60, 6/26, W83, HG564 and HNA99 by using an expression vector. Samples were negatively stained with 1% ammonium molybdate. Some fimbriae appear to be bundled. Bars show 0.1 mm.
(PDF)

Figure S6 **ELISA using unabsorbed antisera and whole-cell sonicates as antigens.** Whole-cell sonicates were coated on ELISA plate as antigens. Antisera of mice immunized with fimbriae from each genotype were used without absorption. Some of the antisera showed substantial titers to some antigens, including the negative control of the fimbriae-deficient mutant. Circles indicate individual serum samples, and horizontal bars indicate means. “Non” is non-immunized mice sera.
(PDF)

Figure S7 **ELISA using unabsorbed antisera and purified FimA fimbriae as antigens.** Purified FimA fimbriae were coated on ELISA plate as antigens. Antisera of mice immunized with fimbriae from each genotype were used without absorption. Circles indicate individual serum samples, and horizontal bars indicate means. “Non” is non-immunized mice sera.
(PDF)

Figure S8 **Phylogenetic tree.** Multiple sequence alignment between FimA fimbriae of *P. gingivalis* strains by ClustalW.
(PDF)

Table S1 Primers for construction of the mfa1-deletion mutant.
(DOC)

Table S2 Primers for construction of the fim cluster-deletion mutant.
(DOC)

Table S3 Primers for fimX, pgmA and fimA cloning.
(DOC)

Table S4 Primers for fimA cloning from each genotype strain.
(DOC)

Table S5 Primers for fimX cloning.
(DOC)
