# Supplementary material for: Porphyromonas gingivalis FimA Fimbriae: Fimbrial Assembly by fimA Alone in the fim Gene Cluster and Differential Antigenicity among fimA Genotypes
Source: PLoS One. 2012 Sep 7;7(9):e43722. doi: 10.1371/journal.pone.0043722 (PMC3436787; doi:10.1371/journal.pone.0043722)
Supplement: Information S1 — DNA sequences analyzed in this study. (DOC) [file pone.0043722.s001.doc]

Supporting Information S2

DNA sequences analyzed in this study. Red letters indicate different nucleotides from the published data in GenBank.

6/26 *fimA orf* (1161 bp)

ACCESSION D17801.1, GI:456504

ATGAAAAAAACAAAGTTTTTCTTGTTGGGACTTGCTGCTCTTGCTATGACAGCTTGTAACAAAGACAACGAGGCAGAACCTGTTACAGAAGGTAATGCTACCATCAGCGTGGTATTGAAGACCAGCAATCCGAATCGTGCTTTTGGAAATGCGGGAGACGAAGCAAAAGTGGCTAAGTTGACCGTAATGGTTTACAAGGGTGAACAGCAGGAAGCCATCAAATCAGCCGAAAATGCGACTAAGGTTGAAAACATCAAATGTAGTGCAGGCCAACGTACGCTGGTCGTAATGGCCAATACGGGTGGAATGGAATTGGCTGGCAAGACTCTTGCAGAGGTAAAAGCATTGACAACTGAACTGACTGAAGGAAACCAAGAGGCTGCAGGGTTGATCATGACAGCAGAGCCTGTTGAGGTAACACTTGTCGCCGGCAATAACTATTATGGTTATGATGGATCTCAGGGAGGTAATCAGATTTCGCAAGATACTCCTCTTGAAATCAAACGTGTTCATGCCCGTATTGCGTTCACCAAGATTGAAGTGACGATGAGCCAGTCTTATGCGAACAAATACAATTTTGCCCCCGAAAACATCTATGCACTTGTGGCTAAAAAGAAGTCTAATCTATTCGGTGCTTCATTGGCAAATAATGATGATGCTTATTTGACTGGTTCTTTGACGACTTTCAACGGAGCTTATACCCCTGCAAACTATACTCATGTCGACTGGTTGGGAAGAGACTTCACAGAGCCTTCCAATAATGCTCCACAAGGTTTCTATGTTTTGGAGAGCACATACGCTCAGAATGCAGGTCTACGTCCTACTATTCTATGTGTAAAAGGCAAGCTGACAAAGCATGATGGTACTCCTTTGAGTTCTGAGGAAATGACAGCTGCATTCAATGCCGGCTGGATTGTTGCAAACAATGATCCTACGACCTATTATCCTGTATTAGTGAACTTTGAGAGCAATAATTACACCTACACAGGTGAGGCTGTTGAGAAAGGAAAAATCGTTCGTAACCATAAATTCGACATCAACCTGACGATCACCGGTCCTGGTACGAATAATCCTGAAAACCCCATTACTGAGTCTGCTAACCTCAACGTTAATTGTGTGGTTGCTGCCTGGAAAGGTGTTGTACAAAATGTTATTTGGTAA

HG564 *fimA orf* (1167 bp)

ACCESSION D17802.1, GI:456506

ATGAAAAAAACAAAGTTTTTCTTGTTGGGACTTGCTGCTCTTGCTATGACAGCTTGTAACAAAGACAACGAGGCAGAACCCATTGTGGAAACTGACGCTACTGTTAGTTTCATAATTAAGAGCGGAGAGGGGCGTGCTGTAGGCGATGGCCTTGCAGATGCCAAGATCACAAAACTCACCGCCATGGTCTATGCAGGTCAAATTCAAGAAGGGATTAAGACAGTGGAAGAGGCCGACGGAGTTCTTAAAGTAGAAGGAATTCCGTGTAAATCAGGAGCCAACCGTGTCCTCGTCGTTGTAGCTAATCACAATTATGAGCTTACCGGTAAAAGTTTGAATGAGGTTGAGGCCTTGACGACTTCTTTGACAGCTGAAAACCAAAATGCCAAAAACTTGATCATGACAGGTAAGTCAGCAGCTTTTACAATCAAGCCGGGCTCCAACCACTATGGCTATCCTGATGGGACTACATCCGACAACCTTGTTTCTGCTGGAACTCCTCTTGCCGTTACTCGCGTGCATGCCGGTATCTCATTCGCAGGAGTAGAGGTAAATATGGCTACACAGTATCAAAACTACTATTCTTTTAACCCAGCTGACGCTAAAATCGCAGCCCTTGTCGCAAAGAAAGATTCTAAGATTTTCGGCAATTCTTTGGTCTCAAACACTAATGCATATTTGTATGGAGTCCAAACGCCTGCCGGTCTTTACACTCCGGATGCTGCAGGAGAAACATACGAATTGGAGGCGTCTTTGAATACGAATTATGCTGTAGGTGCCGGCTTCTATGTGCTGGAAAGTAAATATGATGCAAGCAACGAGCTTCGTCCGACGATCCTTTGTATCTATGGAAAGCTGCTCGATAAGGACGGCAACCCTCTCACGGAACCAGCCTTGACGGATGCTATAAATGCCGGATTCTGCGACGGAGATGGCACGACTTACTATCCGGTATTGGTGAACTATGATGGCAATGGCTACATCTATTCAGGTGCTATTACCCAAGGACAAAACAAAATCGTTCGCAACAACCACTACAAGATTACGCTGAACATCACCGGCCCCGGTACGAATACTCCTGAAAATCCTCAACCGGTACAAGCCAACCTGAATGTTACTTGCCAAGTTACACCTTGGGTTGTTGTTAATCAGGCTGCTACTTGGTAA

HNA99 *fimA orf* (1188 bp)

ACCESSION AB027294.1, GI:6429668

ATGAAAAAAACAAAGTTTTTCTTGTTGGGACTTGCTGCTCTTGCTATGACAGCTTGTAACAAAGACAACGAGGCAGAACCCATTGTGGAAACTGACGCTACTGTTAGTTTCATAATTAAGGCTGGCTCGCCACAACGTGAAACAGAACCCAACAGTCTCCTTGACAGTGATGCCAAAATCACCAAATTGACAGCTATGGTCTATGCAGGTCAAGTTCAAGAAGGAATTAAGACTGTAGAAGATGCTGACAACGTACTCAAAGTTGAAGGTATCAAATGTAAATCAGGAGCAAACAAAGTATTGGTTGTTGTTGCCAATTATGACAAAAATGCAGGTGGAGACGCGATTGACTTTACCGGAAAAACGTTGGATCAGGTAAAAGCTATGACGATCCAACTGACGCAGGACAATCAAAGCGCAAAGTTCTTGATTATGACAGGAGAATCCAACGCTTTTACTATCAAGCCCGGAACAAACTACTATGGCTATCCCGCTGGAACAGGGACTACACAAGACAACCTCATCGAAACTGGCAACGCTCTTAAGGTGACACGTGTTCATGCAGCCATGTCTATCCAAAACGTGACAGTAACGTTCGACCCCCAATACTCAAGCAACTATTATTTTAAGCCGCAAAATGTAGCAGGGTTGATCTGCAAAAAACAATCTAAGATTTTCGGTGCTTCTTTGGATTTTGGCACAGACTACCTCGGTGGCGTAGCTACTACAGCTGCAGCTTATACTCCGACCTCCTACGATAACAACGTAAGCTGGTTGACCAAGCCTTACGCCGCAAAAGCCGGCTTCTATATTATGGAAAGCGTATATCAGGTTGGCAACAATCTTCGTCCTACTATTCTTTGCGTATATGGTAAGCTAAAGAAAACAGAAACACAAGACTTTTCTCAAGAGGAATTGGATGCTGCTGTGGCTGCAGGCTATTGCGATGGCAATGCCATTACGTATTACCCAGTATTGGTTAACTATAATGGTTATGGATATACCTACACTGGAGAGAATACAGGGCTGAACAAAATTCTTCGCAACAACCATTACAAAATCTCATTGACGGTGAAAGGTCCTGGAACGAATACGCCTGAAGGACCTCTGCCTGAAGAAGCTAATCTGAACGTTAACTGTGAGGTTGTTTCTTGGAACGTTGTTAACCAAAGTGCCATTTGGAATTAA
