# Supplementary material for: Porphyromonas gingivalis FimA Fimbriae: Fimbrial Assembly by fimA Alone in the fim Gene Cluster and Differential Antigenicity among fimA Genotypes
Source: PLoS One. 2012 Sep 7;7(9):e43722. doi: 10.1371/journal.pone.0043722 (PMC3436787; doi:10.1371/journal.pone.0043722)

Supporting Information S3

Multiple sequence alignment between FimA fimbriae of *P. gingivalis* strains by ClustalW. Phylogenetic tree is shown in Fig. S7. *, :, . indicate identical amino acids, conservative substitutions and semi-conservative substitutions, respectively


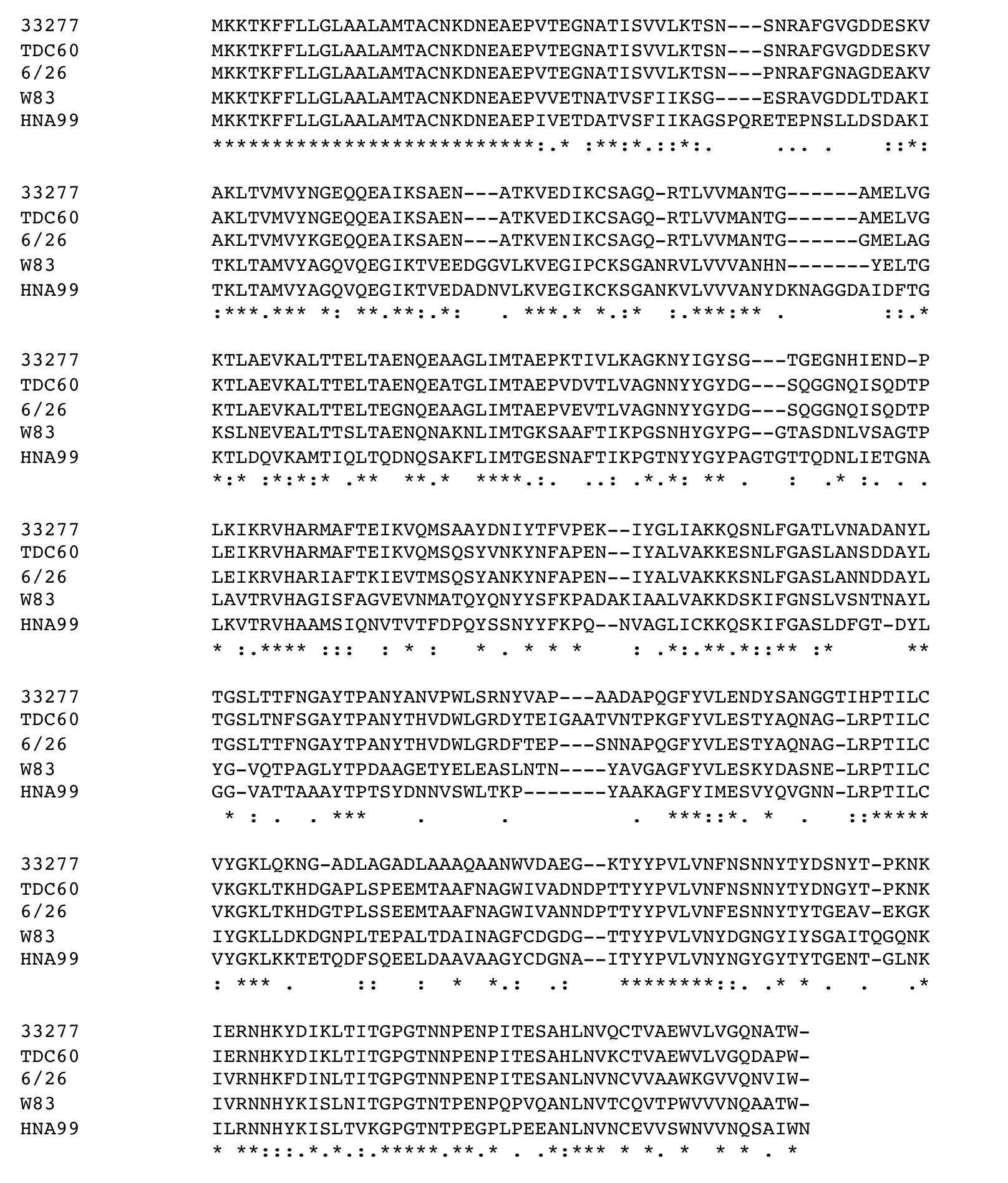

Supplement: Information S2 — Multiple sequence alignment between FimA fimbriae of P. gingivalis strains by ClustalW. (DOC) [file pone.0043722.s002.doc]
