# Supplementary figures and images for: Porphyromonas gingivalis FimA Fimbriae: Fimbrial Assembly by fimA Alone in the fim Gene Cluster and Differential Antigenicity among fimA Genotypes
Source: PLoS One. 2012 Sep 7;7(9):e43722. doi: 10.1371/journal.pone.0043722 (PMC3436787; doi:10.1371/journal.pone.0043722)

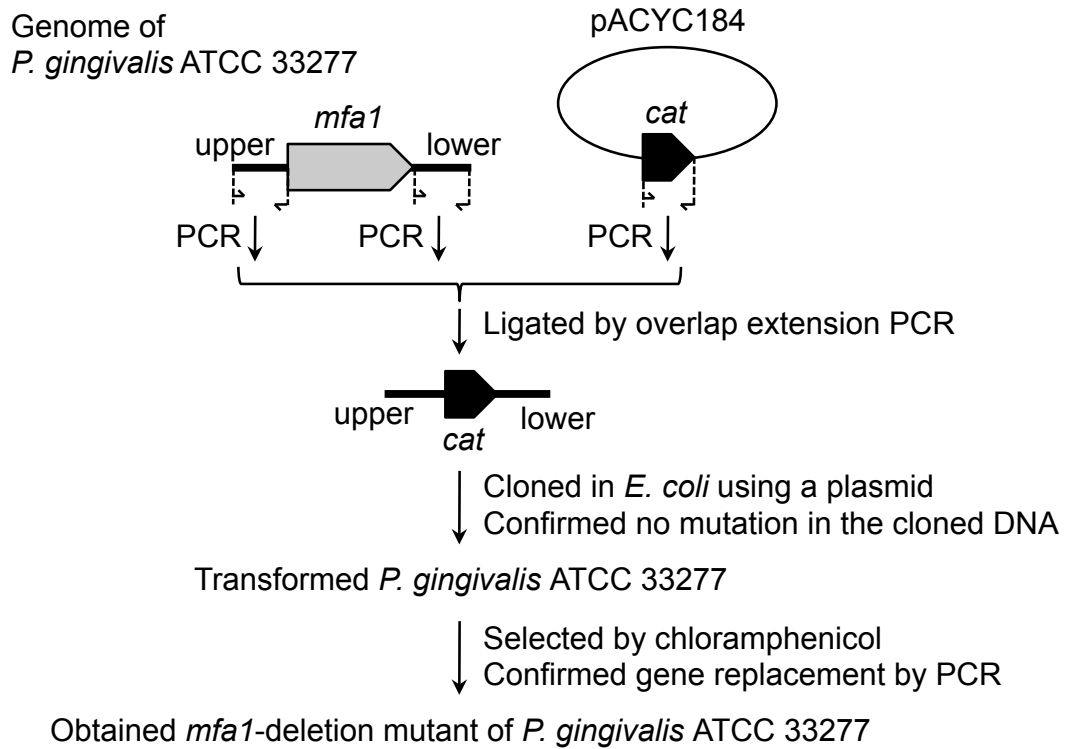

**Figure S1 Construction of a *mfa1*-deletion mutant of *P. gingivalis*.**  
Small arrows show the primers.

Supplement: Figure S1 — Construction of a mfa1- deletion mutant of P. gingivalis . Small arrows show the primers. (PDF) [file pone.0043722.s003.pdf]
