# Supplementary material for: Porphyromonas gingivalis FimA Fimbriae: Fimbrial Assembly by fimA Alone in the fim Gene Cluster and Differential Antigenicity among fimA Genotypes
Source: PLoS One. 2012 Sep 7;7(9):e43722. doi: 10.1371/journal.pone.0043722 (PMC3436787; doi:10.1371/journal.pone.0043722)
Supplement: Figure S2 — Construction of a fim cluster-deletion mutant of P. gingivalis . Small arrows show the primers. ermF-ermB confers erythromycin resistance to P. gingivalis. ermB was previously called ermAM, but the current nomenclature proposed to use ermB (Roberts et al., Antimicrob. Agents Chemother. 1999, 43: 2823–30), therefore we used here ermB. (PDF) [file pone.0043722.s004.pdf]

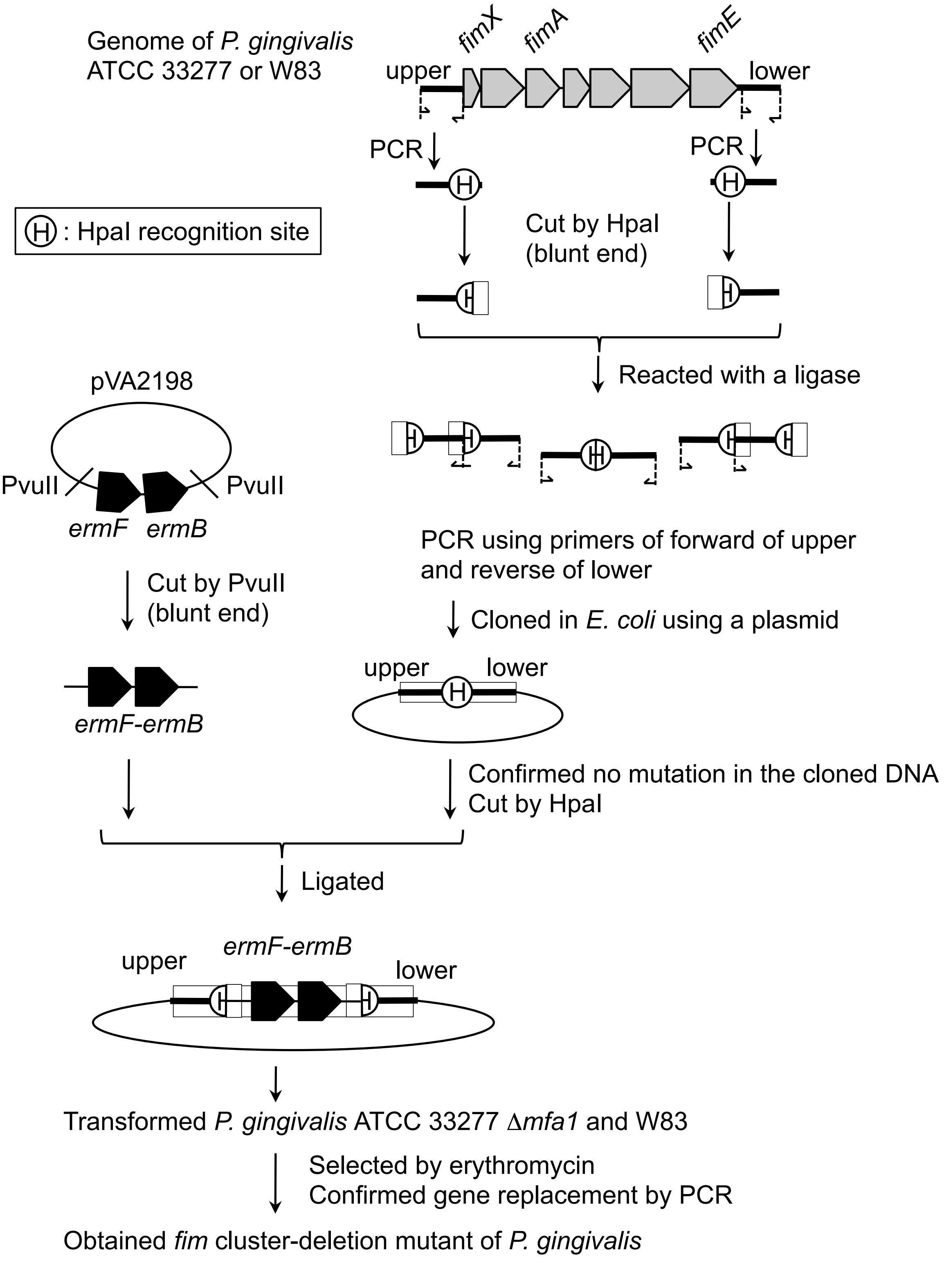

Figure. S2 Construction of a *fim* cluster-deletion mutant of *P. gingivalis.*
Small arrows show the primers. *ermF-ermB* confers erythromycin resistance to *P. gingivalis*. *ermB* was previously called *ermAM*, but the current nomenclature proposed to use *ermB* (Roberts et al., Antimicrob. Agents Chemother. 1999, 43: 2823-30), therefore we used here *ermB* .
