# Supplementary material for: Porphyromonas gingivalis FimA Fimbriae: Fimbrial Assembly by fimA Alone in the fim Gene Cluster and Differential Antigenicity among fimA Genotypes
Source: PLoS One. 2012 Sep 7;7(9):e43722. doi: 10.1371/journal.pone.0043722 (PMC3436787; doi:10.1371/journal.pone.0043722)
Supplement: Figure S3 — Complementary introductions of fim -cluster genes into the fim cluster-deletion mutant of P. gingivalis . fimX-pgmA-fimA, pgmA-fimA, fimX & fimA, or fimA gene were introduced into fim cluster-deletion mutant of P. gingivalis. Small arrows show the primers. tetQ confers tetracycline resistance to P. gingivalis. (PDF) [file pone.0043722.s005.pdf]

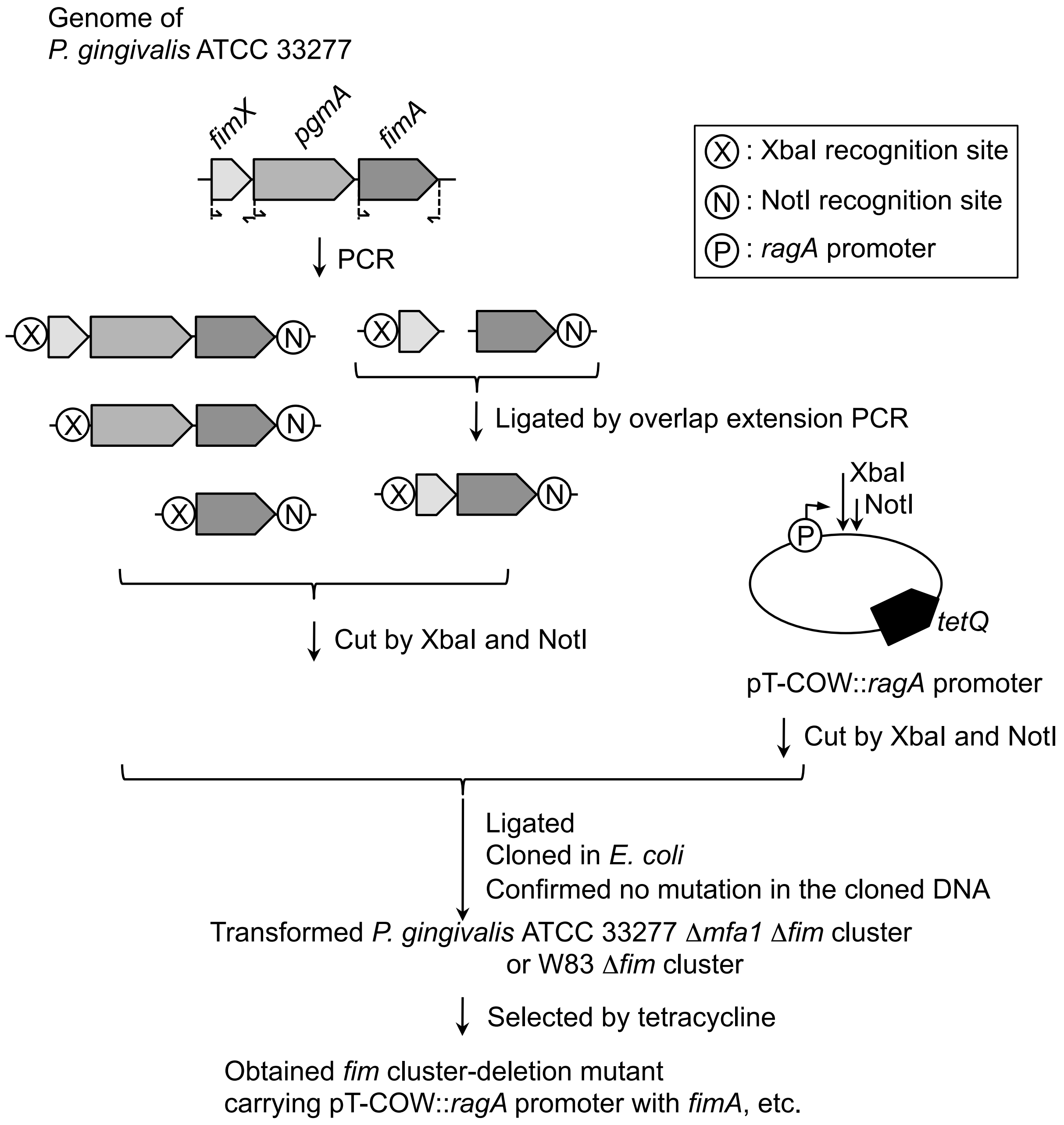

Figure S3 Complementary introductions of *fim*-cluster genes into the *fim* cluster-deletion mutant of *P. gingivalis.*

fimX-pgmA-fimA, pgmA-fimA, fimX & *fimA*, or fimA gene were introduced into *fim* cluster-deletion mutant of *P. gingivalis*. Small arrows show the primers. *tetQ* confers tetracycline resistance to *P. gingivalis.*
