# Supplementary material for: Porphyromonas gingivalis FimA Fimbriae: Fimbrial Assembly by fimA Alone in the fim Gene Cluster and Differential Antigenicity among fimA Genotypes
Source: PLoS One. 2012 Sep 7;7(9):e43722. doi: 10.1371/journal.pone.0043722 (PMC3436787; doi:10.1371/journal.pone.0043722)
Supplement: Figure S4 — Introduction of the fimA gene of each genotype into the fim cluster-deletion mutant of P. gingivalis . The fimA gene from various P. gingivalis strains including ATCC 33277 (type I), TDC60 (type II), 6/26 (type III), W83 (type IV), HG564 (type IV), and HNA99 (typeV) were introduced into fim cluster-deletion mutant of P. gingivalis. Small arrows show the primers. tetQ confers tetracycline resistance to P. gingivalis. (PDF) [file pone.0043722.s006.pdf]

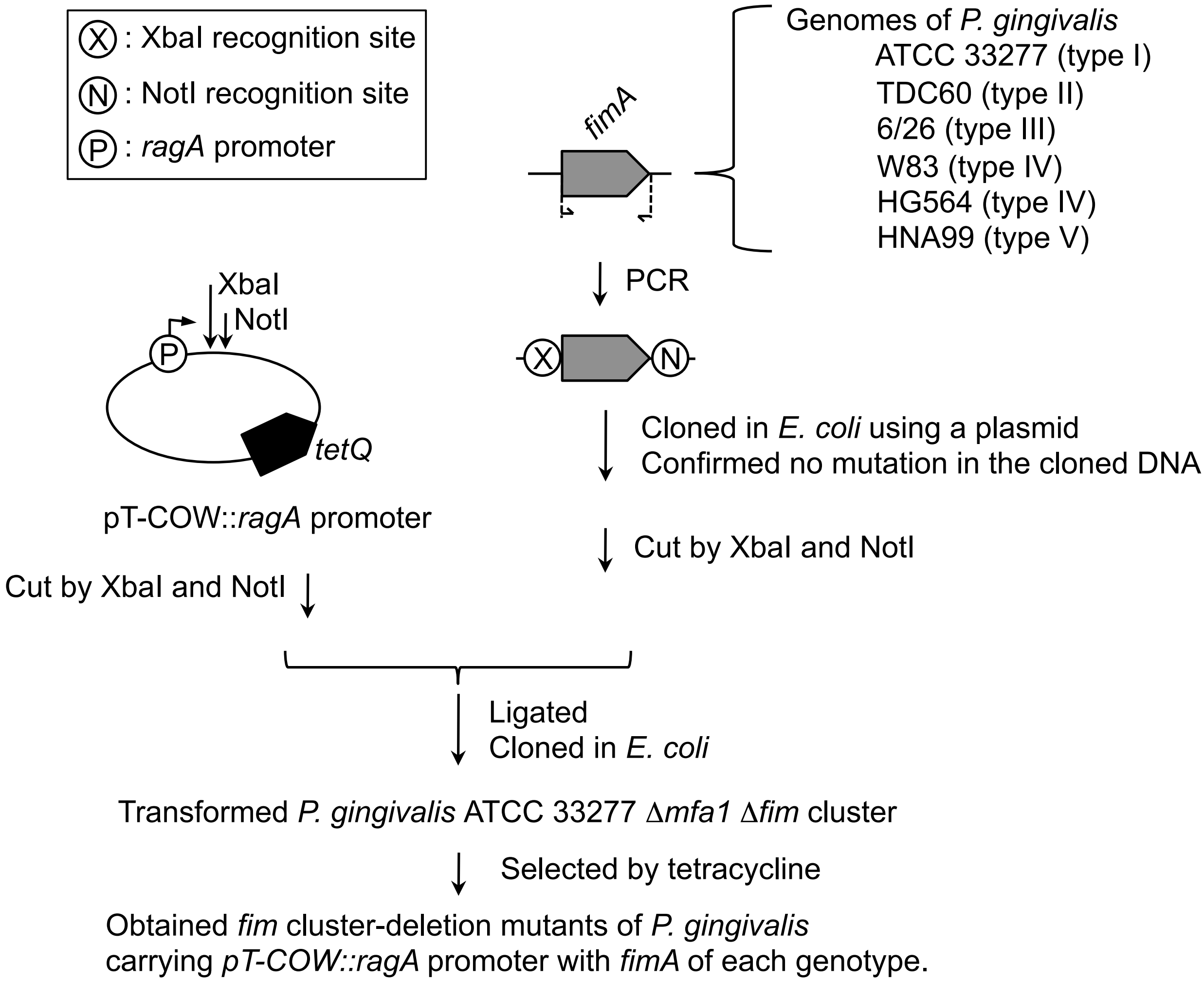

Figure S4 Introduction of the *fimA* gene of each genotype into the *fim* cluster-deletion mutant of *P. gingivalis.*

The *fimA* gene from various *P. gingivalis* strains including ATCC 33277 (type I), TDC60 (type II), 6/26 (type III), W83 (type IV), HG564 (type IV), and HNA99 (typeV) were introduced into *fim* cluster-deletion mutant of *P. gingivalis*. Small arrows show the primers. *tetQ* confers tetracycline resistance to *P. gingivalis.*
