# Supplementary material for: Porphyromonas gingivalis FimA Fimbriae: Fimbrial Assembly by fimA Alone in the fim Gene Cluster and Differential Antigenicity among fimA Genotypes
Source: PLoS One. 2012 Sep 7;7(9):e43722. doi: 10.1371/journal.pone.0043722 (PMC3436787; doi:10.1371/journal.pone.0043722)
Supplement: Figure S5 — Transmission electron microscopic observation of FimA fimbriae purified from complements. FimA fimbriae were purified from P. gingivalis ATCC 33277 Δmfa1 (Native 33277), and P. gingivalis ATCC 33277 Δmfa1 Δfim cluster cells with fimA from 33277, TDC60, 6/26, W83, HG564 and HNA99 by using an expression vector. Samples were negatively stained with 1% ammonium molybdate. Some fimbriae appear to be bundled. Bars show 0.1 mm. (PDF) [file pone.0043722.s007.pdf]

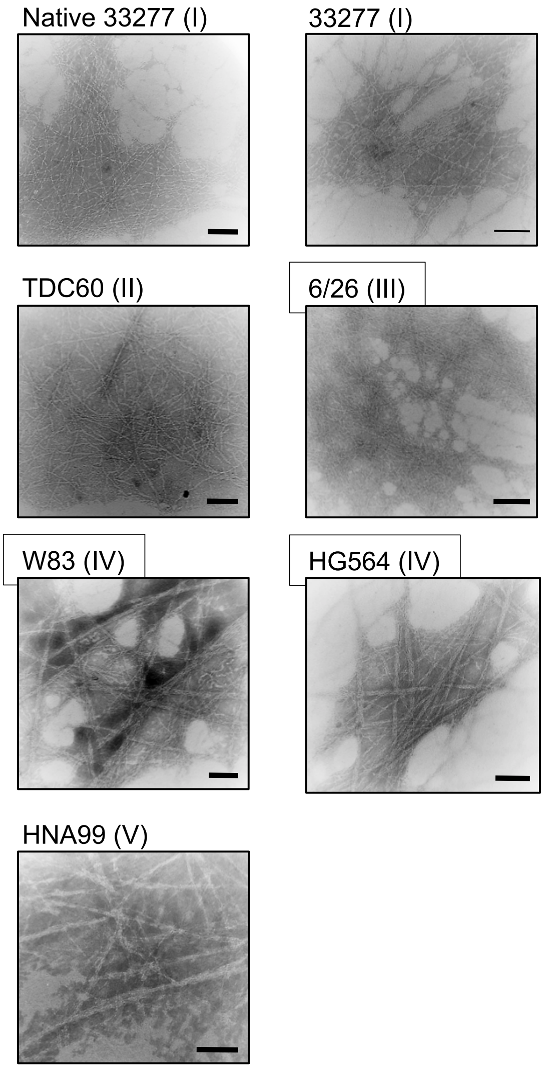

Figure S5 Transmission electron microscopic observation of FimA fimbriae purified from complements.
FimA fimbriae were purified from *P. gingivalis* ATCC 33277 *Δmfa1* (Native 33277), and *P. gingivalis* ATCC 33277 *Δmfa1 Δfim* cluster cells with *fimA* from 33277, TDC60, 6/26, W83, HG564 and HNA99 by using an expression vector. Samples were negatively stained with 1% ammonium molybdate. Some fimbriae appear to be bundled. Bars show 0.1 mm.
