# Supplementary material for: Porphyromonas gingivalis FimA Fimbriae: Fimbrial Assembly by fimA Alone in the fim Gene Cluster and Differential Antigenicity among fimA Genotypes
Source: PLoS One. 2012 Sep 7;7(9):e43722. doi: 10.1371/journal.pone.0043722 (PMC3436787; doi:10.1371/journal.pone.0043722)
Supplement: Figure S6 — ELISA using unabsorbed antisera and whole-cell sonicates as antigens. Whole-cell sonicates were coated on ELISA plate as antigens. Antisera of mice immunized with fimbriae from each genotype were used without absorption. Some of the antisera showed substantial titers to some antigens, including the negative control of the fimbriae-deficient mutant. Circles indicate individual serum samples, and horizontal bars indicate means. “Non” is non-immunized mice sera. (PDF) [file pone.0043722.s008.pdf]

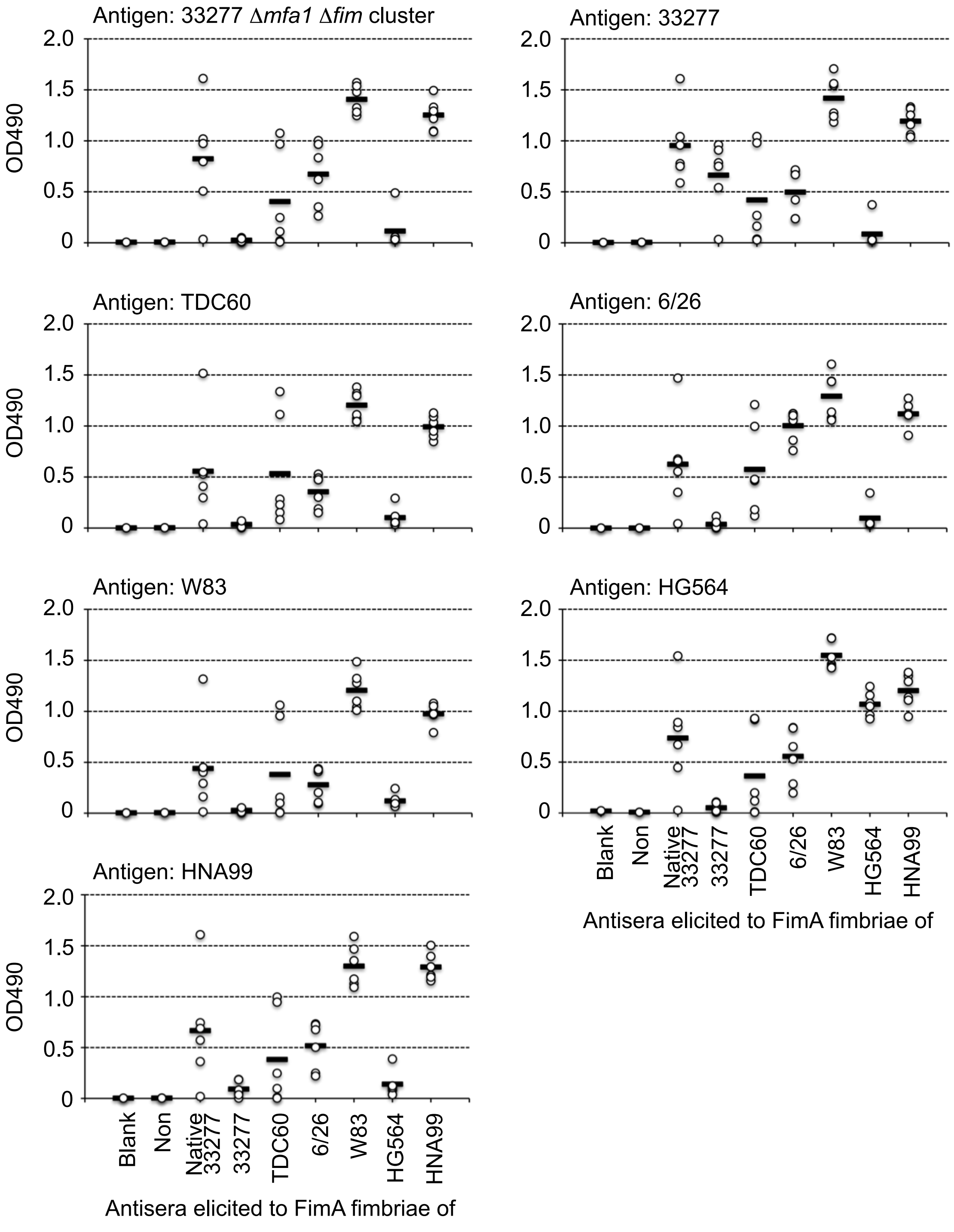

Figure S6 ELISA using unabsorbed antisera and whole-cell sonicates as antigens. Whole-cell sonicates were coated on ELISA plate as antigens. Antisera of mice immunized with fimbriae from each genotype were used without absorption. Some of the antisera showed substantial titers to some antigens, including the negative control of the fimbriae-deficient mutant. Circles indicate individual serum samples, and horizontal bars indicate means. "Non" is non-immunized mice sera.
