# Supplementary material for: Porphyromonas gingivalis FimA Fimbriae: Fimbrial Assembly by fimA Alone in the fim Gene Cluster and Differential Antigenicity among fimA Genotypes
Source: PLoS One. 2012 Sep 7;7(9):e43722. doi: 10.1371/journal.pone.0043722 (PMC3436787; doi:10.1371/journal.pone.0043722)
Supplement: Figure S7 — ELISA using unabsorbed antisera and purified FimA fimbriae as antigens. Purified FimA fimbriae were coated on ELISA plate as antigens. Antisera of mice immunized with fimbriae from each genotype were used without absorption. Circles indicate individual serum samples, and horizontal bars indicate means. “Non” is non-immunized mice sera. (PDF) [file pone.0043722.s009.pdf]

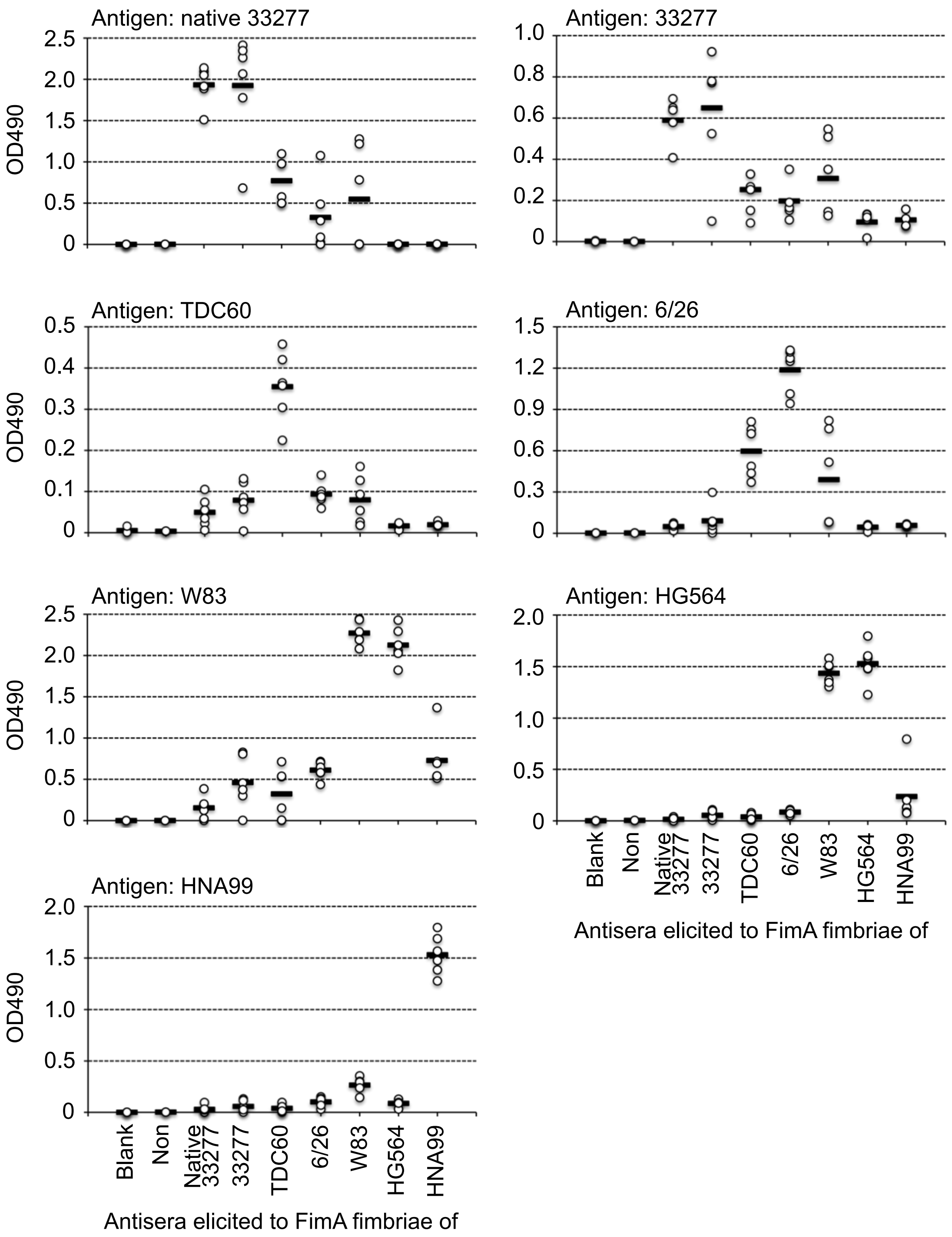

Figure S7 ELISA using unabsorbed antisera and purified FimA fimbriae as antigens. Purified FimA fimbriae were coated on ELISA plate as antigens. Antisera of mice immunized with fimbriae from each genotype were used without absorption. Circles indicate individual serum samples, and horizontal bars indicate means. “Non” is non-immunized mice sera.
