# Supplementary material for: Porphyromonas gingivalis FimA Fimbriae: Fimbrial Assembly by fimA Alone in the fim Gene Cluster and Differential Antigenicity among fimA Genotypes
Source: PLoS One. 2012 Sep 7;7(9):e43722. doi: 10.1371/journal.pone.0043722 (PMC3436787; doi:10.1371/journal.pone.0043722)
Supplement: Figure S8 — Phylogenetic tree. Multiple sequence alignment between FimA fimbriae of P. gingivalis strains by ClustalW. (PDF) [file pone.0043722.s010.pdf]

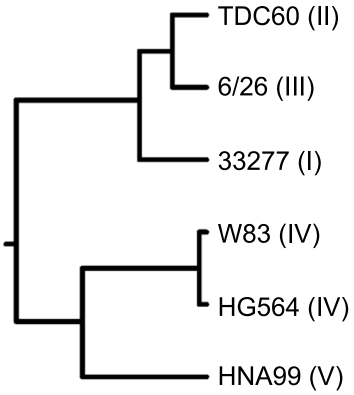

Figure S8 Phylogenetic tree.
Multiple sequence alignment between FimA fimbriae of *P. gingivalis* strains by ClustalW.
