# Supplementary material for: Porphyromonas gingivalis FimA Fimbriae: Fimbrial Assembly by fimA Alone in the fim Gene Cluster and Differential Antigenicity among fimA Genotypes
Source: PLoS One. 2012 Sep 7;7(9):e43722. doi: 10.1371/journal.pone.0043722 (PMC3436787; doi:10.1371/journal.pone.0043722)
Supplement: Table S1 — Primers for construction of the mfa1-deletion mutant. (DOC) [file pone.0043722.s011.doc]

Table S1 Primers for construction of the *mfa1*-deletion mutant.

| Name | Sequence (5’-) | Description |
| --- | --- | --- |
| mfa1 upper F | GACTACATCTCTCGCGAGGG | Forward primer to amplify *mfa1* upper region |
| mfa1 upper R | TCCAGTGATTTTTTTCTCCATAAGCCAAATGTTTAAAAGGATTAATATTAAATTG | Reverse primer to amplify *mfa1* upper region |
| mfa1 lower F | TTACGCCCCGCCCTGCCACTCTTAGCTATTGTAAAATTTTCTTTTTGAGGGTGGG | Forward primer to amplify *mfa1* lower region |
| mfa1 lower R | CTCAACGACAGGACTCGTCGCATACC | Reverse primer to amplify *mfa1* lower region |
| cat F | ATGGAGAAAAAAATCACTGGA | Forward primer to amplify *cat* orf |
| cat R | TTACGCCCCGCCCTGCCACTC | Reverse primer to amplify *cat* orf |
