# Supplementary material for: Porphyromonas gingivalis FimA Fimbriae: Fimbrial Assembly by fimA Alone in the fim Gene Cluster and Differential Antigenicity among fimA Genotypes
Source: PLoS One. 2012 Sep 7;7(9):e43722. doi: 10.1371/journal.pone.0043722 (PMC3436787; doi:10.1371/journal.pone.0043722)
Supplement: Table S2 — Primers for construction of the fim cluster-deletion mutant. (DOC) [file pone.0043722.s012.doc]

Table S2 Primers for construction of the *fim* cluster-deletion mutant.

| Name | Sequence (5’-) | Description |
| --- | --- | --- |
| W83fim cluster upper F | ACGGGATGGAACTTGGAAACAG | Forward primer to amplify *fim* cluster upper region of W83 |
| W83fim cluster upper HpaI R | GTGATTTTCACGTTAACTGTCAACCGAAATTAGACCTAAG | Reverse primer to amplify *fim* cluster upper region of W83, incorporated with HpaI recognition site |
| 33277fim cluster upper F | AAAAATCAGCAGAATCGGAGAAGAAG | Forward primer to amplify *fim* cluster upper region of 33277 |
| 33277fim cluster upper HpaI R | GTGATTTTCACGTTAACGAAGGTGGTGGAAAGCTACTTGGTAG | Reverse primer to amplify *fim* cluster upper region of 33277, incorporated with HpaI recognition site |
| fim cluster lower HpaI F | CCACCTTCGTTAACGTGAAAATCACGAGCGTGAAAAGGAG | Forward primer to amplify *fim* cluster lower region of W83 and 33277 |
| fim cluster lower R | ATCGGACATTCGATTCATCATCTCGG | Reverse primer to amplify *fim* cluster lower region of W83 and 33277, incorporated with HpaI recognition site |

Underlines indicate restriction-enzyme recognition sequences.
