# Supplementary material for: Porphyromonas gingivalis FimA Fimbriae: Fimbrial Assembly by fimA Alone in the fim Gene Cluster and Differential Antigenicity among fimA Genotypes
Source: PLoS One. 2012 Sep 7;7(9):e43722. doi: 10.1371/journal.pone.0043722 (PMC3436787; doi:10.1371/journal.pone.0043722)
Supplement: Table S3 — Primers for fimX, pgmA and fimA cloning. (DOC) [file pone.0043722.s013.doc]

Table S3 Primers for *fimX*, *pgmA* and *fimA* cloning.

| Name | Sequence (5’-) | Description |
| --- | --- | --- |
| fimA XbaI F | AACAAATCTAGAATGAAAAAAACAAAGTTTTTCTTGTTGGGAC | Forward primer to amplify *fimA* of 33277, incorporated with XbaI recognition site |
| fimA NotI R | GTTTGAGCGGCCGCTTACCAAGTAGCATTCTGACCAACGAGAAC | Reverse primer to amplify *fimA*, *pgmA* and *fimX* of 33277, incorporated with NotI recognition site |
| pgmA XbaI F | CAGACATCTAGAATGAAACAGAGTCGCCATATCATCC | Forward primer to amplify a DNA fragment from *pgmA* to *fimA* of 33277, incorporated with XbaI recognition site |
| fimX XbaI F | CACAAATCTAGAATGAAAAGGAAAACACGATTGCTGATTATC | Forward primer to amplify a DNA fragment from *fimX* to *fimA* of 33277, incorporated with XbaI recognition site |
| fimA+fimX F | CATCATTAAGTAAATGGGAAAAGATTAGATTTTTAGAAAAC | Forward primer to amplify *fimA* of 33277 to construct DNA fragment, fused with 3’ terminal of *fimX* |
| fimX+fimA R | AATCTTTTCCCATTTACTTAATGATGTATATAAGCGAAAGTG | Reverse primer to amplify *fimX* of 33277 to construct DNA fragment, fused with 5’ terminal of *fimA* |

Underlines indicate restriction-enzyme recognition sequences.
