# Supplementary material for: Porphyromonas gingivalis FimA Fimbriae: Fimbrial Assembly by fimA Alone in the fim Gene Cluster and Differential Antigenicity among fimA Genotypes
Source: PLoS One. 2012 Sep 7;7(9):e43722. doi: 10.1371/journal.pone.0043722 (PMC3436787; doi:10.1371/journal.pone.0043722)
Supplement: Table S4 — Primers for fimA cloning from each genotype strain. (DOC) [file pone.0043722.s014.doc]

Table S4 Primers for *fimA* cloning from each genotype strain.

| Name | Sequence (5’-) | Description |
| --- | --- | --- |
| fimA XbaI F | AACAAATCTAGAATGAAAAAAACAAAGTTTTTCTTGTTGGGAC | Forward primer to amplify *fimA* of all strains, incorporated with XbaI recognition site |
| fimA NotI R | GTTTGAGCGGCCGCTTACCAAGTAGCATTCTGACCAACGAGAAC | Reverse primer to amplify *fimA* of 33277, incorporated with NotI recognition site |
| TDC60fimA NotI R | TAGACAAACTATGAAAGCGGCCGCGTCGTTTGACGGGTCGATTACC | Reverse primer to amplify *fimA* of TDC60, incorporated with NotI recognition site |
| 6/26 fimA NotI R | TTTTGAGCGGCCGCTTACCAAATAACATTTTGTACAACACCTTTC | Reverse primer to amplify *fimA* of 6/26, incorporated with NotI recognition site |
| W83fimA NotI R | GTTTGAGCGGCCGCTTACCAAGTAGCAGCCTGATTAACAACAAC | Reverse primer to amplify *fimA* of W83 and HG564, incorporated with NotI recognition site |
| HNA99fimA NotI R | TTTCTTGCGGCCGCTTAATTCCAAATGGCACTTTGGTTAACAAC | Reverse primer to amplify *fimA* of HNA99, incorporated with NotI recognition site |

Underlines indicate restriction-enzyme recognition sequences.
