# Supplementary material for: Porphyromonas gingivalis FimA Fimbriae: Fimbrial Assembly by fimA Alone in the fim Gene Cluster and Differential Antigenicity among fimA Genotypes
Source: PLoS One. 2012 Sep 7;7(9):e43722. doi: 10.1371/journal.pone.0043722 (PMC3436787; doi:10.1371/journal.pone.0043722)
Supplement: Table S5 — Primers for fimX cloning. (DOC) [file pone.0043722.s015.doc]

Table S5 Primers for *fimX* cloning.

| Name | Sequence (5’-) | Description |
| --- | --- | --- |
| fimX BamHI F | ACACAAGGATCCGATGAAAAGGAAAACACGATTGCTGATTATC | Forward primer to amplify *fimX*, incorporated with BamHI recognition site |
| fimX XhoI R | ATAAATCTCGAGTTACTTAATGATGTATATAAGCGAAAGTG | Reverse primer to amplify *fimX*, incorporated with XhoI recognition site |

Underlines indicate restriction-enzyme recognition sequences.
